# Microbial- and Plant-Derived Bioactive Peptides and Their Applications against Foodborne Pathogens: Current Status and Future Prospects

**DOI:** 10.1155/2024/9978033

**Published:** 2024-04-29

**Authors:** Anowar Khasru Parvez, Fatema Tuz Jubyda, Mohammed Ayaz, Amily Sarker, Nabila Haque, Md. Shahriar Khan, Taslin Jahan Mou, Md. Atikur Rahman, Md. Amdadul Huq

**Affiliations:** ^1^Department of Microbiology, Jahangirnagar University, Savar, Dhaka, Bangladesh; ^2^Institute of Biodiversity, Aquatic Geomicrobiology, Friedrich Schiller University Jena, Jena, Thuringia, Germany; ^3^Department of Food and Nutrition, College of Biotechnology and Natural Resource, Chung-Ang University, Anseong 17546, Gyeonggi-do, Republic of Korea

## Abstract

Bioactive peptides (BAPs) obtained from plants and microbes have been thoroughly explored and studied due to their prophylactic properties. The use of BAPs seems to be a promising substitute for several currently available antibiotics because of their antimicrobial properties against foodborne pathogens. BAPs have several other useful properties including antitumor, antihypertensive, antioxidant, antiobesity, and antidiabetic activities. Nowadays, scientists have attempted to recombinantly synthesize bioactive peptides to study their characteristics and potential uses, since BAPs are not found in large quantities in nature. Many pathogenic microorganisms including foodborne pathogens are becoming resistant to various antibiotics. To combat these pathogens, scientists are working to find novel, innovative, and safe antimicrobial agents. Plant- and microbe-based BAPs have demonstrated noteworthy antimicrobial activity against a wide range of pathogenic microorganisms, including foodborne pathogens. BAPs can kill pathogenic microorganisms by disrupting membrane integrity, inhibiting DNA and RNA synthesis, preventing protein synthesis, blocking protein activity, or interacting with certain intracellular targets. In addition, the positive effect of BAP consumption extends to gut microbiota modulation and affects the equilibrium of reactive oxygen species in the gut. This article discusses recombinant BAPs, BAPs generated from plants and microbes, and their antimicrobial applications and modes of action for controlling foodborne pathogens.

## 1. Introduction

Bioactive peptides have been thoroughly researched for their health advantages and possible applications as nutraceuticals and functional food ingredients [1]. Emerging antibiotic resistance (ABR) related to foodborne illnesses harms both human and animal populations as well as the economy and human health suffers greatly from bacterial resistance which prompts food to deteriorate and causes health problems. Finding antibiotic substitutes that can reduce health risks and the consequences of numerous foodborne illnesses is now vital [2]. If BAPs are employed as food additives or preventative measures, they exhibit potential as safe food supplements with a variety of health benefits and BAPs are less likely than antibiotics to cause bacteria to evolve resistance [3].

BAPs are often embedded in the structure of large proteins and are cleaved to become active. They consist of short amino acid (aa) sequences (2–20 aa) that provide beneficial health facilities, such as, in the form of controlling foodborne pathogens [4]. They can be derived from microbes, plants, and animal tissues. In addition, food processing (cooking, fermentation, and ripening) or microbial enzyme maturation may produce BAPs. Products including milk, cheese, yogurt, sausage, eggs, soybeans, soy milk, chia, rice bran, peas, flaxseed, mushrooms, and cauliflower are considered richer sources of BAPs [5]. Many classes of BAPs are currently available for purchase chemically or recombinantly [6].

There are a variety of plant-derived BAPs such as puroindolines, lipid transfer proteins, thionins (*α*/*β*, *γ*), glycine-rich peptides, hevein-like peptides, plant defensins, and knottin-like peptides. Numerous classes of stems, roots, seeds, flowers, and leaves have all been found to contain BAPs and they show activity against both phytopathogens and bacteria that are harmful to humans [7]. Microbes also produce a large variety of biologically active peptides such as pediocins, nisin, enterocin, propionicin, gramicidin, endolysins, and apicidin. These peptides are derived from viruses, bacteria, and fungi. They protect organisms from invasive bacteria, viruses, protozoa, and fungi by disrupting the membrane or metabolic processes [8].

Peptides can regulate a number of vital physiological functions such as hormones. Their positive health impacts include cardiovascular disease reduction, immunomodulatory, antihypertensive effects, mineral binding, chelating action, and anticoagulant, antioxidative, and antimicrobial properties. Besides, they are in charge of the flavor of the food as well as preventing the activity of enzymes that cause diseases to develop [8]. Some BAPs through their antimicrobial activity protect mammals from various foodborne pathogens as well as their direct impact on the shelf life of prepared foods has made them highly desirable in this industry [9]. These peptides can change the biological processes of pathogens, such as the growth of cells and the formation of cell membranes. They are thought to work by opening up channels or pores in bacterial membranes, which prevent anabolic processes, and alter gene expression and signal transmission while encouraging angiogenesis [10]. Another immunomodulatory result, that may halt the spread of foodborne illnesses caused by the foodborne pathogen, *Listeria monocytogenes* can lead to severe listeriosis and develop resistance to antibiotics [11]. Therefore, as an antimicrobial peptide, BAP can kill pathogens through a variety of actions such as interrupting membrane integrity, hindering DNA and RNA synthesis, preventing protein synthesis, and acting on particular intracellular targets [12, 13]. Besides, the positive effect of BAP by consuming it also includes gut microbiota regulation. Recent research has linked the prevention and treatment of neurodegenerative illnesses such as Parkinson's disease, Alzheimer's disease, and dementia to gut microbiome modifications supported by BAPs [14, 15].

Food safety and human health are seriously compromised by foodborne pathogens. They are responsible for various life-threatening diseases in humans. Moreover, a large number of these foodborne bacteria are developing antibiotic resistance on a daily basis [11]. Therefore, it is essential to develop safe and effective antimicrobial agents to control these foodborne pathogens (e.g., viruses, bacteria, and parasites) for food safety as well as to protect human health. Bioactive antimicrobial peptides could be a good option for the development of novel antimicrobial agents against foodborne pathogenic microorganisms such as *Escherichia coli, Salmonella, Listeria, Cyclospora, Campylobacter,* and *Shigella*. The naturally derived BAPs possess significant antiviral activity against the hepatitis virus along with other viruses as these BAPs can inhibit viral entry into the host cells and interfere with host-specific interactions. From this perspective, this review highlights currently known plant- and microbes-derived bioactive BAPs as well as recombinant BAPs with their mechanisms of action for controlling foodborne pathogens and their prospects.

## 2. BAPs from Different Sources

### 2.1. Plant-Derived BAPs

Plants produce a large variety of biologically active peptides such as thionins (*α*/*β*, *γ*), puroindolines, lipid transfer proteins, plant defensins, glycine-rich peptides, hevein-like peptides, knottin-like peptides, and homologs of MBP-1, which work against bacterial and fungal pathogens (Table 1). There are five types of thionins: grain endosperms that contain purothionins, which are type I thionins; *α*-hordothionin and *β*-hordothionin are type II thionins that are found in *Pyrularia pubera* leaves and nuts; ligatoxin A and viscotoxins are examples of type III thionins; and crambin and hellothionin D peptides belong to groups IV and V, respectively [7, 13, 16]. Plant defensins are small, cysteine-rich, cationic antimicrobial peptides containing conserved 3D structures comprising one *α*-helix and three antiparallel *β*-strands. *γ*-Hordothionin is a member of plant defensins and Ah-AMP1, Ct-AMP1, Rs-AFP1, PhD1, and Hs-AFp1 are examples of plant defensins [7, 45]. Most plant defensins fall into one of the three categories based on the quantity and location of cysteine residues inside the molecules: hevein-type, knottin-type, and thionin-type [50].

### 2.2. Microbe-Derived BAPs

Microbe-derived BAPs are found in viruses, bacteria, and fungi. These peptides are classified based on their sources, structural characteristics, amino-acid-rich content, and activities. Viral BAPs are phage proteins containing virion-associated peptidoglycan hydrolases, depolymerases, lysins, and holins called enzybiotics. Phage-tail complexes and phage-encoded lytic factors are two types of phage BAPs. Bacterial BAPs are produced by both Gram-positive and Gram-negative bacteria. Gram-positive bacterial BAPs are classified as ribosomally-produced BAPs known as bacteriocin and nonribosomally or enzymatically-produced BAPs [8]. There are two types of bacteriocins: lantibiotics and nonlantibiotics (lantibiotics contain unnatural amino acid lanthionine) [68]. Gram-negative bacterial bacteriocins are grouped into colicins, microcins, colicin-like bacteriocins, and phage-tail-like bacteriocins. Defensins and peptaibol are the two types of fungal BAPs. Peptaibol contains the name in combination with peptide, *α*-aminoisobutyrate, and amino alcohols. Microbe-derived BAPs and their potential antimicrobial applications are shown in Table 2.

### 2.3. Recombinant BAPs

Once the peptide sequence is revealed, it can be synthesized chemically or by utilizing recombinant DNA technologies. Hydrolysis by enzymes is a simple production process, but it takes time and requires sophisticated purifying methods. Furthermore, the yield of natural proteins is restricted by their extremely low BAP content. Even though chemical synthesis is the most mature technology for peptide production, the necessity of toxic reagents for their chemical production and lack of specificity are severe drawbacks. On the other hand, recombinant DNA technology uses fewer chemicals and makes the synthesis of these proteins simpler with high yield and purity without any environmental impact [113, 114].

According to multiple studies, recombinant production of BAPs can be categorized into two classes, depending on bioactive peptide gene expression in a particular expression system, either *in vivo* or *in vitro* [6]. In the *in vivo* expression method, the targeted peptide gene is linked to another known carrier protein gene to make the purification simple and to be able to produce a mass amount of the necessary peptide. For instance, ecallantide and desirudin peptides are expressed in yeast [115, 116]. On the other hand, the *in vitro* expression method, which is a cell-independent system, has the benefit of rapid production of the desired outcome though it is not cost-effective [117]. However, the recent focused method is the engineering of BAPs, due to peptide flexibility and effectiveness. For example, engineered insulin plays a crucial role in type II diabetes with a longer effect than own insulin [118].

One study investigated the recombinant synthesis of the BAP, GIISHR (Gly-Ile-Ile-Ser-His-Arg) with notable antioxidant activity from the muscle of the flawless smooth-hound (*Mustelus griseus*) [119]. Antioxidant peptides isolated from spotless smooth-hound exhibited good scavenging activities and protected H_2_O_2_-induced HepG2 cells from oxidative stress by increasing the levels of catalase, superoxide dismutase, glutathione peroxidase, and glutathione reductase along with decreasing the content of malonaldehyde [4]. The peptide had a strong ability to neutralize hydroxyl, ABTS (2,2′-Azino-bis-(3-ethylbenzotiazoline-6-sulfonic acid)), and superoxide radicals [119]. At the beginning, the strain as host such as *E. coli* needs to be selected and then the expression vector is designed. Tricine-SDS-PAGE and western blot analysis are employed to assess the amount of expression of the recombinant protein [120], which is followed by trypsin digestion and purification of the peptide [121]. Then, the purified peptide was analyzed by liquid chromatography and their activity was tested by different assays [4].

An antagonistic peptide, Turgincin A is recombinantly produced by the *Pichia pastoris* strain. This recombinant peptide prevents the growth of all bacteria in the pork meat while preserving the meat's color [21]. Another recent recombinant fusion peptide, CpsA-CpsC-L-ACAN consists of three parts: CpsAo CpsC, the enzymes that produce *Streptococcus agalactiae* capsules, serine, and glycine, a linker, and ACAN, an anticancer component. As a result, this peptide shows good antimicrobial performance against *E. coli* and *Staphylococcus aureus* [22].

## 3. Antimicrobial Applications of BAPs against Foodborne Pathogens

Both the health of people and the economy are at stake due to the rise in the frequency of foodborne diseases. The presence of harmful bacteria, viruses, fungi, parasites, and toxins in food that is contaminated has been linked to more than 200 various diseases [122]. Due to this, the application of preservatives is required in a wide variety of foods to ensure safety while preserving the product's quality and sensory qualities. In addition, as it was already indicated, efforts are continually made to find natural antimicrobials in order to keep up with the customer demand [123]. In this case, the application of BAPs becomes necessary.

Plant-derived BAPs having antimicrobial properties have been identified and described in a wide range of structural and functional ways up to this point. From a library of cDNA derived from Mexican avocado fruits, PaDef was found and isolated. It is a peptide with defensin-like properties. As PaDef exhibits antibacterial properties against *S. aureus* and *E. coli*, it can be used to heal foodborne infections [124]. In a different investigation, four closely similar cysteine-rich peptides with antifungal and antibacterial properties were extracted and described from the grain of Impatiens species balsamina [125]. In addition, these cysteine-rich peptides showed high activity against enteric pathogens such as *S. aureus, E. coli,* and *Salmonella* yet showed no cytotoxicity toward human cells. Plants have also been found to contain 2S albumin proteins, another family of AMPs. Pa-AFP-1 was discovered to effectively suppress the growth of filamentous fungi, including *Trichoderma harzianum, Colletotrichum gloeosporioides, Aspergillus fumigatus,* and *Fusarium oxysporum,* which were isolated from passion fruit [126, 127]. The next compound is CaThi, an isolated and described thionin-like peptide from chili. According to reports, CaThi is effective against a variety of pathogenic bacteria, including *Candida albicans, F. solani, C. tropicalis,* and *S. cerevisiae* [128, 129].

The main enzyme for photorespiration and photosynthesis in plants as well as in other living things is known as ribulose-1,5-bisphosphate carboxylase/oxygenase (RuBisCO), which is also the most prevalent protein in the world. RuBisCO is a desirable and long-term resource for BAPs [130]. RuBisCO 407 large subunit-derived peptides ELAAAC (f454-459), and MDN (472-474), as well as the original hydrolysate and portions generated by hydrolyzing RuBisCO with pepsin, all exhibited antimicrobial activity against Gram-positive (*L. innocua, Micrococcus luteus,* and *Bacillus subtilis*) and Gram-negative (*E. coli*) microorganisms [131]. Unquestionably, a revolutionary method for producing BAPs with a variety of positive health effects involves the fermentation by microorganisms of protein from different sources. The BAPs produced by microbial fermentation can be further purified without hydrolysis, and it is less expensive than using enzymes [8].

Gram-positive lactic acid bacteria (LAB) are the source of a wide range of bioactive substances, such as fatty acids, hydrogen peroxide, short-chain peptides, and fatty acids. The importance of LAB in the food and beverage business extends far beyond the manufacture of fermented foods because many of these substances have a bioprotective action against infections and degrading agents [132, 133]. *Lactiplantibacillus plantarum*, a LAB that is known to produce antimicrobial peptides, has been examined in various investigations to determine whether it has the capacity to inhibit significant foodborne pathogens [134–136]. Research also showed that *L. plantarum*, cell-free supernatant, and isolated bacteriocins from this strain enhance direct inhibition. In addition, it has been noted that BAPs produced from *L. plantarum* have the properties of proteolyzing milk proteins [137–139]. *S. aureus, L. monocytogenes, E. coli,* and *S. Typhimurium* are only a few of the Gram-positive and Gram-negative foodborne pathogenic organisms that have been found to be inhibited by *L. plantarum* fermented camel's milk [137, 140, 141]. In addition, a significant amount of low-molecular-weight antimicrobial peptides were found among which 32 of these peptides came from milk proteins in the most effective fraction [138]. *Lactobacillus casei* ATCC 334 producing BAP-P1, P2, and P4 in the fermented fat kenaf grain had a strong antibacterial action against *S. Typhimurium, E. coli, Pseudomonas aeruginosa, S. aureus,* and other microorganisms [142]. Bioactive antimicrobial peptides also exhibit antivirulence property activity against foodborne pathogens at subinhibitory concentrations. Various BAPs have bactericidal effects on biofilm formation and can eradicate infections in animal models [143].

Nanoantimicrobials are frequently utilized to treat bacterial infections directly. Nanoantimicrobials, also known as nanoantibiotics, are nanoparticles exhibiting antimicrobial activity or enhancing the activity of encapsulated antimicrobial agents. Chitosan nanoparticles and peptides, known as CNMs, are outstanding new antibacterial medications that are a promising alternative to antibiotics for use against harmful bacteria. By using a digestive epithelial cell framework, the role of CNMs was assessed in the prevention of *E. coli* O157 infection. CNMs exhibited good bactericidal effects against *E. coli* O157, according to antibacterial activity testing [144].

**Figure 1:**
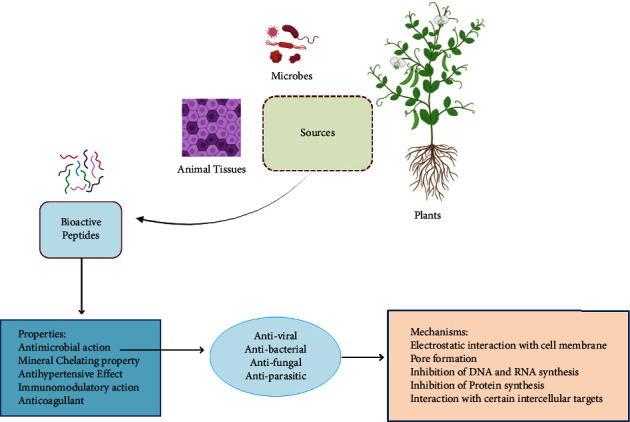
Schematic illustration of BAPs and their potential antimicrobial applications against foodborne pathogens.

Both Gram-positive and Gram-negative bacteria, including*, E. coli, L. monocytogenes, S. aureus, P. aeruginosa,* and *S. enterica* were inhibited by the antibacterial activity of BAPs generated by *Bifidobacterium lactis* BB-12 and *Lactobacillus acidophilus* LA-5 in milk model medium and their combination cultures [145].  Figure 1 shows the antimicrobial applications of bioactive peptides.

The primary biotechnological tool for producing Brewer's or Baker's biomasses, which are mostly used in the production of fermented foods and beverages, is *Saccharomyces cerevisiae*. The *S. cerevisiae* precursor proteins enolase II and glyceraldehyde-3-phosphate dehydrogenase are notable because they released BAPs having antimicrobial properties with the highest scores. In particular, protein-sealing antibacterial peptides that exhibit broad-spectrum activity and may prevent cytotoxicity while also reducing the emergence of microbial resistance ought to be considered a reliable and year-round source for next-generation bioactive substances. *S. cerevisiae* biomass is a food-grade product that is sold and consumed globally [146].

## 4. Mode of Action of BAPs against Foodborne Pathogens

**Figure 2:**
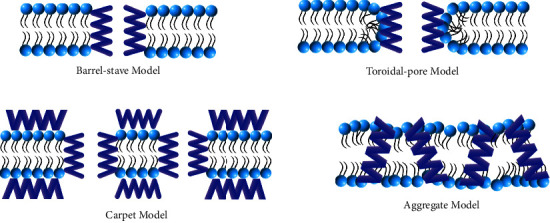
Models of membrane-cavity formation. BAPs' direct bactericidal mechanism involves their interaction with negatively charged membranes, which leads to increased cell membrane permeability, rupture of the cell membrane, or the release of internal contents, and ultimately, cell death. The formation of membrane pores may involve the toroidal-pore, aggregate, barrel-stave, and carpet models, respectively. The hydrophobic sections of peptides enter the phospholipid membrane mix with the internal hydrophobic portions of the phospholipid bilayer, leaving the hydrophilic portions exposed to the outside.

**Figure 3:**
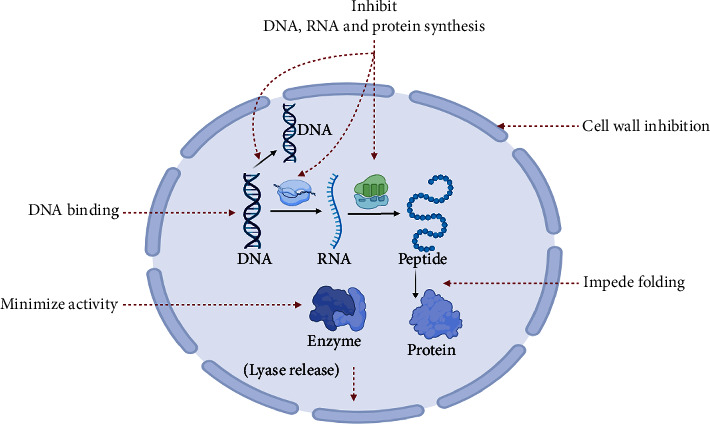
The intracellular modes of action of BAPs. The penetration of BAPs into the cell protoplasm, allowing their interactions with intracellular activities, causes the inhibition of cell wall synthesis, DNA, RNA, and protein synthesis, protein folding, and enzyme activity. Another bactericidal mechanism of certain BAPs is to activate autolysin to destruct cell structures.

Nomura's 1967 identified two mode of actions of biopeptides [147]. One method showed how bacteria might attach to peptides, creating pores or holes in their cell walls. Another method proposed how contact strength damages the chemical and biological structures of afflicted cells [148]. The interaction of peptides with sensitive cells can occur in two ways: (1) cell wall receptors bind to the peptide molecules and it does not affect the physiological makeup of cells or (2) the impacted cells suffer biological and chemical harm [149]. The interaction between positively charged BAPs and the mannan of fungi, lipoteichoic acid of Gram-positive bacteria, and LPS of Gram-negative bacteria is characterized by a significant affinity [150]. Figures 2 and 3 show the possible antimicrobial mechanisms of BAPs.

### 4.1. Alteration of Outer Membrane Permeability

BAPs can enter the membrane and perform intracellular functions or permeate the membrane and cause intracellular contents to leak [147, 150]. The interaction of peptides with cell walls is primarily influenced by conformational change and the peptide-lipid ratio [151–154]. In an aqueous solution, alpha-helical peptides attach to the negatively-charged lipid membrane and change its disorganized structure. The stability of disulfide bond bridges in *β*-sheet peptides is attributed to their lack of conformational changes during interaction with the plasma membrane [155]. The peptide-lipid ratio is another important factor since low values lead peptides to be located parallel to the bacterial cell membrane [156, 157].

Some speculative models of membrane-cavity formation have been put forth, including the barrel-stave, toroidal-pore, carpet, and aggregate models (Figure 2) [147]. In the barrel-stave model, when more peptide binds to the membrane, aggregation and conformational modification take place, leading to a shift in the local phospholipid head groups and thinning of the membrane [68]. During penetration into the phospholipid bilayer, the hydrophilic sections of the peptide helixes face inside, whereas the hydrophobic portions of the *β*-sheet and *α*-helical peptides are near the cell membrane phospholipid. The core lumen is formed by paralleling many helical molecules [155]. Unlike the barrel-stave model, the toroidal-pore model involves peptide helices penetrating the cell membrane and interacting with lipids to construct the toroidal-pore complexes. High quantities of locally-gathered peptides cause lipid molecules to bend, which allows both the lipid head groups and peptides anchored inside the core of the lipid to move [68]. While the electrostatic impact of peptides and anionic membrane is essential in the carpet model, significant peptide concentrations must be present to produce micelles and damage the microbial membrane [155]. While the concentration of peptide crosses the threshold, clusters of peptides coat the membrane and break it like a surfactant. In the hydrophobic core of the membrane, neither channel development nor peptide insertion takes place. This action is strong enough to cause cell death by partial or total lysis of the cell membrane [147].

According to the aggregation model, lipids and peptides are compelled to assemble a micelle of the peptide-lipid complex when peptides attach to the anionic cytoplasmic membrane [158]. In comparison to the carpet concept, peptides, lipids, and water in cellular channels allow ions and subcellular components to flow. Cell death results from leakage. These channels might also assist in the transfer of peptides into the cytoplasm, where they can function. This mechanism explains why peptides can act on intracellular substances in addition to the cytoplasmic membrane, which is their primary target [159]. Unlike cationic peptides, the mechanisms underlying anionic peptides are still unknown. Maximin H5's antimicrobial effect against *S. aureus* has been believed to underlie membrane dissolution [160]. Other reported modes of action also include membrane destruction. For instance, clavanin A embraces the *α*-helical peptide membrane permeation mode in neutral pH [161]. However, it causes cell death in slightly acidic pH by acting on membrane proteins that keep a constant pH gradient. An essential step for disrupting microbial surfaces is the LPS anchored in the bacterial pathogen's outer membrane [162]. The vital role of the synchronized opening movements of the LPS transport (Lpt) *β*-taco domain and *β*-barrel of the LPS transport protein has been shown to facilitate the insertion of LPS into the bacterial surface. Since thanatin stabilizes the *β*-taco domain, LPS cannot be transported to the cell surface [162].

#### 4.1.1. Alteration of the Intracellular Mechanism of Action

Buforin II, a BAP, containing 21 amino acids, displays antibacterial action against a variety of microorganisms [163]. It shares the same sequence as a piece of the protein called histone H2A, which directly engages nucleic acids [163]. Previous studies have shown that buforin II has the capacity to bind to DNA and RNA, as well as penetrate lipid vesicles *in vitro*, hence potentially affecting the permeability of the membrane [163]. PR-39, a BAP, isolated from the small intestine of pigs and high in proline and arginine, was discovered to quickly permeate the outer membrane of *E. coli* [164]. After entering the cytoplasm, PR-39 interferes with the synthesis of proteins, leading to the breakdown of proteins essential for the synthesis of DNA. Consequently, this disruption impairs the process of DNA synthesis. Usually, the proline-enriched BAPs attach to ribosomes and obstruct protein production [165].

According to reports, peptides stop a bacterial intracellular enzyme from working. The bacterium heat shock protein DnaK, which was isolated from protein lysates of *E. coli* and shown to be selectively bound by pyrrhocoricin, was demonstrated by the Otvos' group [166]. The same team went on to show in a subsequent investigation that pyrrhocoricin prevented DnaK from acting as an ATPase [167]. It was first discovered that human neutrophil peptide-1 could enter both the inner and outer membranes of *E. coli* and inhibit the making of the bacteria's DNA, RNA, and proteins [168]. The deadly event, it should be noted, seems to be inner membrane permeabilization. The enzymatic activities of D-Ala-D-Ala ligase and alanine racemase are essential for the biosynthesis of D-Ala-D-Ala dipeptide, a key component of lipid II, that serves as a precursor molecule in the formation of peptidoglycan. The antibacterial activity of bacteria may be restricted by cycloserine via the inhibition of D-Ala-D-Ala ligase and alanine racemase [169].

As BAPs exhibit antibacterial activity by membrane or nonmembrane-mediated action either by increasing membrane permeability or pore formation leading to the leakage of intracellular contents, or penetration into the membrane to exert intracellular actions without targeting specific molecules/pathways, it is unlikely to develop bacterial resistance to BAPs [147, 150]. The maximal H5 engages in interactions with bacteria through its N-terminal helical peptide, while the aspartate residues primarily serve a minimal function. As a result of their separation from the membrane surface, they play an important structural role. The hydrogen bonds created during the acetylation of the N- and C-terminal ends of the peptide are what stabilize its -helix structure [170]. Despite cell membrane disintegration and intracellular efflux, the anionic peptide Xlasp-p1 displays wide antibacterial activity against Gram-positive and Gram-negative bacteria [171].

A milk-derived peptide (AMP SSSEESII from *α*_s2_-casein) has been shown in many studies to be able to prevent the development of *M. luteus, L. innocua, E. coli,* and *S. enteritidis.* In casein, a different bioactive peptide called IKHQGLPQE reduced the number of harmful microorganisms that are often prevalent in newborn formula [172]. *B. subtilis, S. aureus, S. pneumoniae, E. coli, P. aeruginosa, S. dysenteriae,* and *S. typhimurium* have all been reported to be strongly inhibited by the GLSRLFTALK peptide [173]. Mackerel byproducts *E. coli* and *Listeria innocua* were both suppressed by SIFIQRFTT [174]. Antioxidant activities [175] and immunomodulation [176] can make BAPs better alternatives for antibiotics [172]. Several haemoglobin-derived peptides possess cytotoxic properties against *S. Enteritidis, S. saprophyticus, S. simulants, B. cereus, E. coli, M. luteus, E. faecalis, L. innocua,* and *S. sonnei.* [177, 178].

## 5. Conclusions and Future Prospects

Several studies have demonstrated the significance of BAPs and their applicability in the pharmacological and pharmaceutical fields. They may also be used in crop development and cosmetology, though to a lesser level [179–183]. These bioactivities, especially the antibacterial potential, can be advantageous to the agri-food sector. This business is constantly in need of the creation of effective and secure substitutes for preservatives and food additives. As a result of the advent of antibiotic resistance, the abuse of antibiotics in livestock farming is additionally a significant worldwide public health issue. Researchers are trying to develop unique, safe, and efficient antimicrobial agents [184–188]. The use of BAPs with antimicrobial capacity appears to be a favorable strategy for concerns relating to both food safety and animal growth promotion [189–191]. With demonstrated action against significant bacteria such as *B. subtilis, L. monocytogenes, E. coli, V. parahaemolyticus, P. aeruginosa, S. aureus, K. pneumoniae,* and *S. enterica*, the use of BAPs produced from dietary proteins against foodborne pathogens has a lot of potential. When evaluating the application of BAPs having antimicrobial properties, BAPs as food additives or drugs, it is important to keep in mind that the majority of studies have been conducted *in vitro*, and further research is required to assess the *in vivo* combinations and how they interact with food substrates [192]. Another problem is that BAPs may lose their bioactivity as a result of interactions with other food matrix constituents, food manufacturing, or the intestinal environment. Therefore, when developing functional foods containing these peptides, it is necessary to assess the impact of food manufacturing conditions on the biological activity as well as the availability of these peptides. In addition, it is necessary to examine how these peptides interact with other ingredients once they have been added to the food matrix. It is possible to consider the controlled delivery systems such as microparticulate, nanoemulsion, and nanostructured lipid carriers or chemical changes such as the cyclization of the structures of BAPs that are vulnerable to digestive enzymes or thermal treatment [184]. The feasibility of using controlled delivery methods, such as microparticulate, nanoemulsion, and nanostructured lipid carriers, or implementing chemical modifications, such as cyclization of bioactive peptide structures, to protect against the effects of digestive enzymes or heat treatment may be contemplated [193]. BAPs are intriguing compounds with a wide range of uses because of their antioxidant, anticancer, antihypertensive, antihyperpigmentation, anti-inflammatory, antidiabetic, intestine-modulating, hypocholesterolemic, and antibiotic properties, along with others. BAPs can modulate the composition of gut microbiota facilitating the proliferation of those with antiobesity effects that exert antiobesity effects by controlling the energy balance of the host and food intake along with suppressing the growth of proobesity gut bacteria. Future studies should therefore concentrate on encouraging the commercial manufacturing of stable, plant- and microbe-based BAPs that may be used in a variety of food matrices without impairing food quality or bioavailability along with controlling foodborne pathogens. Opportunities and difficulties are constantly interconnected, and sufficient scientific evidence supports the idea that BAPs produced from plant materials and microbial sources may exhibit a variety of biological and functional features, highlighting their enormous potential in the food industry as well as for controlling the emergence of foodborne pathogens in the future.

## Figures and Tables

**Table 1:** Plant-derived bioactive peptides and their potential antimicrobial applications.

Name of the peptide	Plant source	Target pathogen	Mode of action	References
Thionins	Wheat, barley, oats, rice, rye, mistletoe, oil nut, and Abyssinian cabbage	Bacteria	Pore formation, ion channel formation, cell membrane disruption, and protein synthesis inhibition	[13]
		Gram-positive or Gram-negative bacteria such as *Pseudomonas*, *Xanthomonas*, *Agrobacterium*, *Erwinia*, and *Corynebacterium*		

Purothionin	Wheat	Bacteria	Interfere with DNA synthesis by inhibiting the activity of the enzyme ribonucleotide reductase, and also inhibit the activity of *β*-glucuronidase	[13, 16]
		*Pseudomonas solanacearum*, *Xanthomonas phaseoli*, *Xanthomonas campestris*, *Erwinia amylovora*, *Corynebacterium flaccumfaciens*, *Corynebacterium michiganense*, *Corynebacterium poinsettiae*, *Corynebacterium sepedonicum,* and *Corynebacterium fascians*		

WAMP_1a and WAMP_1b	Seeds of *T. kiharae*	Fungi	Growth inhibition and induced destruction	[17, 18]
		*Fusarium solani*, *Fusarium oxysporum,* and *Helminthosporium sativum*		
		Bacteria		
		Gram-positive		
		*Clavibacter michiganense,* Gram-negative		
		*Pseudomonas syringae*		
		*Erwinia carotovora*		

Lc-Def	Lentil	Fungi	Electrostatic interaction with anionic lipid components of fungal membranes	[18]
		*Aspergillus niger, Aspergillus versicolor, Botrytis cinerea, Fusarium culmorum, Fusarium solani,* and *Neurospora crassa*		

AFP-J	Potato tuber (*Solanum tuberosum* cv. L Jopung)	Fungi	Inhibit serine protease activity	[12, 19]
		Trichosporon *beigelii, S. cerevisiae,* and *Candida albicans*		
		Bacteria		
		*Staphylococcus aureus, Listeria monocytogenes,* and *Escherichia coli*		

Potide-G	Potato tubers	Bacteria	Suppressed proteolytic activity of trypsin, chymotrypsin, and papain	[12]
		*Staphylococcus aureus, Listeria monocytogenes, Escherichia coli,* and *Clavibacter michiganensis* subsp. *michiganense*		
		Fungi		
		*C. albicans* and *R. solani*		
		Virus Potato virus Y		

PKPI and PPI-I	Potato sprout	Fungi	Inhibit fungal protease, spore germination, hyphal elongation, and the development of necrotic lesions	[12]
		*Botrytis cinerea*		

Potato pseudothionin solanum tuberosum 1 (Pth-St1)	Potato tubers	Bacteria	Bind to the membrane receptor, chelation of Ca^2+^, and consequently, pores are being formed	[12, 20, 21]
		*Clavibacter michiganensis* and *Pseudomonas solanacearum*		
		Fungi		
		*F. solani*		

PKI1 and PPI3B2	Potato	Fungi	Fungal proteases affect spore germination, hyphal elongation, and development of necrotic lesions	[22]
		*Botrytis cinerea*		

PLPKI	Potato	*Phytophthora infestans* and *Rhizoctonia solani*	Inhibited the activity of extracellular proteases	[23]

PSPI-21 and PKSI	Potato tubers	Fungi	Serine proteinases inhibitor (affect the growth of oomycete mycelium and fungal mycelium) induces complete destruction of oomycete zoospores and partial destruction of fungal macroconidia	[24]
		*Phytophthora infestans* and *Fusarium culmorum*		

Snakins (SN1 and SN2)	Potato	Bacteria	Rapid aggregation of both Gram-positive and Gram-negative bacteria	[12, 25]
		*C. michiganensis* subsp. *Sepedonicus, Ralstonia solanacearum,* and *R. meliloti*		
		Fungi		
		*B. cinerea, Fusarium oxysporum f. sp. Conglutinans, F. solani, Bipolaris maydis, Aspergillus flavus,* and *Colletotrichum graminicola*		

2S albumin-like protease inhibitor	Barley seeds	Fungi	Permeabilize fungal membranes	[19, 26]
		*Alternaria brassicicola, Botrytis cinerea, Fusarium culmorum, Fusarium oxysporum f.sp. lycopersici,* *Pyricularia oryzae,* and *Verticillium dahlia*		

Trypsin and chymotrypsin inhibitors	Cabbage leaves	Fungi	Blocked the synthesis of chitin in the cell wall, and weakened the fungal hyphae, thus inducing the leakage of intracellular contents from susceptible fungal species	[19, 27]
		*Botrytis cinerea* and *Fusarium solani*		

ZmESR-6	Kernels of maize	Bacteria	Inhibit protein synthesis and block ion channels	[28, 29]
		*Clavibacter Michiganensis, Xanthomonas campestris,* and *Rhizobium meliloti*		
		Fungi		
		*Fusarium oxysporum f.sp. Conglutinans, Fusarium oxysporum f.sp.lycopersici,* and *Plectosphaerella cucumerina*		

Fabatin	Broad bean	Bacteria	Insertion into the plasma membranes of bacterial cells, leading to depolarization of the membrane and cell lysis	[29–31]
		*Escherichia coli, Enterococcus hirae,* and *Pseudomonas aeruginosa*		

VaD1	Azuki bean	Bacteria	Inhibit protein synthesis	[29, 32]
		*Staphylococcus epidermis, Xanthomonas campestris pv. vesicatoria,* and *Salmonella typhimurium*		
		Fungi		
		*Fusarium oxysporum,* *Fusarium oxysporum,* and *Trichophyton rubrum*		
So-D2 and So-D7	Spinach	Bacteria	Damage the cell wall via repression of gene expression or via restricting bacterial replication, causing cell lysis	[29, 33]
		*Clavibacter* *sepedonicus* and *Ralstonia solanacearum*		

Tu-AMP1 and Tu-AMP2	*Tulipa gesneriana*	Bacteria	Positively charged proteins interact with negatively charged membrane phospholipids, following a membrane permeability modification	[21, 29]
		*Erwinia carotovora* subsp. *Carotovora, A. tumefaciens, Clavibacter michiganensis,* and *Curtobacterium flaccumfaciens pv. oortii*		

Pp-AMP 1 and Pp- AMP 2	Japanese bamboo shoots	Bacteria	Binds to specific phospholipids and cause the exposure of toxicity, resulting in cationic imbalance	[21]
		*E. carotovora, A. radiobacter, A. rhizogenes, C. michiganensis,* and *C. flaccumfaciens*		

MtDef5	*Medicago truncatula*	Bacteria	Permeabilizes the plasma membrane, translocates into the cells of this bacterial pathogen and binds to DNA, membrane permeabilization, and fungal growth arrest	[29, 34]
		*Xanthomonas campestris pv. Campestris*		
		Fungi		
		*Fusarium graminearum* and *Neurospora crassa*		

Tad1	Winter wheat	*Pseudomonas cichorii*	Unknown	[29]

OsDef7 and OsDef8	Rice *Oryza sativa*	Bacteria	Interact with the negatively charged phospholipids on the bacterial membrane surface and fungal membrane destabilization	[29, 35]
		*Xanthomonas oryzae, pv. oryzae, X. oryzae pv. oryzicola, Erwinia carotovora* subsp. *atroseptica, Pseudomonas aeruginosa,* and *Dickeya dadantii*		
		Fungi		
		*Helminthosporium oryzae* and *Fusarium oxysporum f.sp*. *cubense*		

PvD1	Seeds	Fungi	Oxidative damage related to the induction of ROS and NO production, cytoplasmic fragmentation, formation of multiple cytoplasmic vacuoles, and membrane permeabilization in the cells of this organism	[36, 37]
		*C. albicans*		
		Protozoa		
		*Leishmania amazonensis*		

Limenin	*Phaseolus limensis*	Bacteria	Membrane collapse by interacting with lipid molecules on the bacterial cell surface, inhibiting the translation of fungi. HIV-1 reverse transcriptase inhibition	[29, 38, 39]
		*Mycobacterium phlei, Proteus vulgaris, Bacillus megaterium,* and *Bacillus subtilis*		
		Fungi		
		*Botrytis cinerea, Fusarium oxysporum,* and *Mycosphaerella arachidicola*		
		Virus		
		HIV-1		

Ct-AMP1	*Clitoria ternatea*	Fungi	Cause a reduction in hyphal thickness and an apparent collapse of the plasma membrane leading to an apparent fragmentation of the cytoplasm	[29, 40]
		*Botrytis cinerea, Cladosporium sphaerospermum, Fusarium culmorum Leptosphaeria maculans, Penicillium digitatum, Trichoderma viride, Septoria tritici,* and *Verticillium albo-atrum*		

J1-1	*Capsicum annuum*	Bacteria	Binds with phosphoinositides (PIs) and PA	[29]
		*Pseudomonas aeruginosa*		
		Fungi		
		*Fusarium oxysporum* and *Botrytis cinerea*		

MsDef1	*M. sativa*	Fungi	Ion channel blocking and hyperbranching	[41]
		*F. graminearum*		

HaDEF1	*Sunflower*	Fungi	Membrane permeabilization and apoptosis	[41]
		*O. Cumana*, *O. Ramosa*, *S. cerevisiae*, and *A. brassicicola*		

Fa-AMP1 and Fa-AMP2	Buckwheat seeds	Bacteria	Disruption of microbial membranes and phospholipid liposomes, an interaction with a specific receptor as an ion channel or a sphingolipid	[21, 29]
		*Erwinia carotovora* subsp. *carotovora, Agrobacterium tumefaciens, Clavibacter michiganensis, and Curtobacterium flaccumfaciens pv. oortii*		
		Fungi		
		*Fusarium oxysporum* and *Geotrichum candidum*		

*α*-Hordothionins	Barley	Bacteria	Interacting electrically with fungal lipid bilayer and linking to the membrane surface, leading to permeabilization and disruption of the membrane organization	[21, 42]
		*Clavibacter michiganensis* subsp. *michiganensis* and *Xanthomonas campestris* pv. *vesicatoria*		

Pa-AMP-1	Pokeweed seeds	Fungi	Interact with the phospholipids of cell membranes, resulting in the inhibition of fungal growth	[42, 43]
		*Alternaria panax* and *Fusarium* sp.*, Rhizoctonia solani*		

Cp-thionin II (*γ*-thionins)	Cowpea seeds	Bacteria	Insertion into the plasma membranes of bacterial cells, leading to depolarization of the membrane, cell lysis, and permeabilization of the hyphae, leading to leakage and granulation of the plasma membrane, and increased generation of reactive oxygen species (ROS) causes fungal growth inhibition	[29, 30, 44]
		*Pseudomonas syringae, Staphylococcus aureus,* and *Escherichia coli*		
		Fungi		
		*F. culmorum*		

Dm-AMP1	*Dahlia merckii*	Fungi	Causes a reduction in hyphal thickness and an apparent collapse of the plasma membrane leading to an apparent fragmentation of the cytoplasm	[29, 40]
		*Botrytis cinerea, Cladosporium sphaerospermum, Fusarium culmorum* *Leptosphaeria maculans, Penicillium digitatum* *Trichoderma viride, Septoria tritici* *Verticillium albo-atrum*		

Hs-AFP1	*Heuchera sanguinea*	Fungi	Causes germ tubes and hyphae to swell and form multiple hyphal buds, membrane permeabilization, ROS, and apoptosis	[40, 41]
		*Botrytis cinerea, Cladosporium sphaerospermum, Fusarium culmorum,* *Leptosphaeria maculans, C. albicans, C. krusei, A.flavus, Penicillium digitatum, Trichoderma viride, Septoria tritiei,* and *Verticillium albo-atrum*		

Rs-AFP2	Radishes	Fungi	Causes germ tubes and hyphae to swell and form multiple hyphal buds, membrane permeabilization, ROS, apoptosis, inhibit cell growth, and ion flux	[40, 41]
		*Botrytis cinerea* *Cladosporium sphaerospermum, Fusarium culmorum* *Neurospora crassa* *Leptosphaeria maculans, Penicillium digitatum Trichoderma viride* *Septoria tritici* *Verticillium albo-atrum*		

Hc-AFP	*Heliophila coronopifolia*	Fungi	Hyperbranching, fungal tip swelling, increased granulation of hyphae and spores, as well as hyphal and spore, and membrane permeabilization disruption	[41]
		*Botrytis cinerea*		
		*Fusarium solani*		

Ah-AMP1	Horse chestnut	Fungi	Reducing hyphal thickness and collapse of the plasma membrane causes an apparent fragmentation of the cytoplasm	[29, 40]
		*Botrytis cinerea, Cladosporium sphaerospermum,* *Fusarium culmorum,* *Leptosphaeria maculans,* *Penicillium digitatum* *Trichoderma viride* *Septoria tritici, and* *Verticillium albo-atrum*		

NaD1 *γ*-thionin-like protein	*Nicotiana alata*	Fungi	Interacting with the cell wall causes the destruction of internal membrane integrity by membrane permeabilization and targets internal organelles by inducing the development of reactive oxygen species (ROS) and fungal cell death	[37, 41, 45]
		*Leptosphaeria maculans, V. dahlia, Thielaviopsis basicola*, *Aspergillus nidulans, C. albicans, C. neoformans, C. gattii,* and *Fusarium oxysporum*		

BCP-2 alpha thionin	Barley grain	Fungi	Bind to glucosylceramides and sphingolipids, leading to fungal cell lysis	[21, 44, 46]
		*Botrytis cinerea*		
		*Trichoderma viride*		

Mo-CBP_2_ (chitin-binding protein)	Seeds	Fungi	Increased the cell membrane permeabilization and produce reactive oxygen species, have DNase activity	[47]
		*Candida albicans, C. parapsilosis, C. krusei,* and *C. tropicalis*		

Mo-CBP_3_	Seeds	Fungi	Inhibited spore germination and mycelial growth induced the production of ROS and caused disorganization of both the cytoplasm and the plasma membrane leading to cell death	[48]
		*Fusarium solani, F. oxysporum, Colletotrichum musae,* and *C. gloeosporioides*		

Cy-AMP1	Cycad seeds	Fungi	Bind to chitin of fungus surface	[49, 50]
		*F. oxysporum*		
		*G. candidum*		

Lunatusin	Chinese lima bean	Bacteria	Causes membrane collapse by interacting with lipid molecules on the bacterial cell surface, inhibits mycelial growth of fungi, and inhibits HIV-1 reverse transcriptase protein-protein inhibition	[16, 38, 49]
		*Bacillus megaterium, B. subtilis, P. vulgaris,* and *Mycobacterium phlei*		
		Fungi		
		*Fusarium oxysporum*, *Mycosphaerella arachidicola,* and *Botrytis cinerea*		
		Virus		
		HIV-1		

Vulgarinin	Haricot beans	Bacteria	Cell membrane disruption, growth inhibition, and death of bacteria, interaction with phosphorylinositol containing sphingolipids or glycosylceramides cause subsequent fungal cell death, and inhibit HIV-1 reverse transcriptase, protease, and integrase	[16, 38, 51]
		*Mycobacterium phlei*, *Bacillus megaterium*, *B. subtilis, P. vulgaris*		
		Fungi		
		*Botrytis cinerea, Fusarium oxysporum, Physalospora piricola,* and *Mycosphaerella arachidicola*		
		Virus		
		HIV-1		

Hispidalin	*Benincasa hispida*	Bacteria	Amphipathicity and cationic charge of peptide facilitates the peptide attachment and insertion into the bacterial membrane to create transmembrane pores resulting in membrane permeabilization. Fungal hyphae growth inhibition	[16, 52]
		*S. enterica,* *S. aureus,* *E. coli,* and *P. aeruginosa*		
		Fungi		
		*A. flavus,* *F. solani,* *C. geniculate,* and *P. chrysogenum*		

(Cg24-I)	*Carica candamarcensis*, *C. papaya,* and *Cryptostegia grandiflora*	Fungi	Inhibition of mycelia growth and spore germination	[16, 53]
		*F. solani*, *R. solani,* and *F. oxysporum*		

CpLP cysteine-like proteases	*Calotropis procera*	Fungi	Fungal growth inhibition, production of ROS lead to oxidative stress, loss of cell function, and ultimately cell death by apoptosis or necrosis	[54]
		*Colletotrichum gloeosporioides, Fusarium oxysporum,* *Fusarium solani,* *Rhizoctonia solani,* *Neurospora sp.,* and *Aspergillus Niger*		

IbAMP1 plant defensin	Seeds	Bacteria	Increase permeability to the cell membrane, permitting efflux of ATP and interfering with intracellular molecular processes (DNA, RNA, and protein synthesis)	[55, 56]
		*E. coli* O157: H7 and *Staphylococcus aureus*		

WAMPs (hevein-like AMPs)	Wheat	Fungi	Cell wall/membrane disruption, the peptide penetrates through the fungal cell walls and interferes with fungal growth by binding or cross-linking the newly-synthesized chitin chains, penetrating into the fungal hyphae and localized at the septum and hyphal tips, resulting in hyphal tip burst and leakage of the cytoplasmic constituents. Active against fungal metalloproteases	[57, 58]
		*C. cucumerinum, A. alternata* *F. oxysporum,* and *B. sorokiniana*		

AX (cysteine-rich proteins)	Sugar beet leaves	Fungi	Reduction of hyphal growth	[59]
		*C. beticola*		

Ay-AMP	*Amaranthus hypochondriacus* seeds	Fungi	Degrades chitin of the fungal cell walls and accumulates at septa and hyphal tips by the union to the fungus cell wall chitin, inhibiting the growth	[60]
		*Candida albicans, Trichoderma* sp.*, Fusarium solani, Penicillium chrysogenum,* *Geotrichum candidum, Aspergillus candidus, Aspergillus. ochraceus,* and *Alternaria alternata*		

Pn-AMPs (hevein-type)	Seeds of morning glory	Fungi	Penetrated very rapidly into fungal hyphae and localized at septum and hyphal tips of fungi, which caused the burst of hyphal tips. The burst of hyphae resulted in disruption of the fungal membrane and leakage of the cytoplasmic materials	[61]
		*Botrytis cinerea, Phytophthora parasitica, Fusarium oxysporum, Rhizoctonia solani,* and *Saccharomyces cerevisiae*		

GAFP (hevein-type)	Ginkgo biloba	Fungi	Burst of hyphal tips increased hyphal membrane permeabilization	[7]
		*Fusarium graminearum, Fusarium moniliforme, Pellicularia sasakii Ito,* and *Alternaria alternata*		

Ns-D1 and Ns-D2	*Nigella sativa* seeds	Fungi	Inhibited hyphal growth	[7, 62]
		*Aspergillus niger,* *Fusarium oxysporum,* *Fusarium graminearum,* *Fusarium culmorum,* *Bipolaris sorokiniana,* and *Botrytis cinerea*		

PINA and PINB (puroindoline)	Wheat	Fungi	Interactions with cellular membranes and ion channel formation in the membranes	[7]
		*Alternaria brassicicola,* *Ascochyta pisi,* *Botrytis cinerea,* *Verticillium dahliae,* *Fusarium culmorum,* and *Cochliobolus heterostrophus*		

Ha-AP10 (lipid transfer proteins)	Sunflower seeds (*Helianthus annuus*)	Fungi	Membrane permeabilization by electrostatic interaction with anionic membrane phospholipids induces liposome leakage and permeabilization of fungal spores	[55, 63]
		*Fusarium solani*		

WjAMP1 (hevein-like AMPs)	Leaves of *Wasabia japonica* L.	Bacteria	Peptide binding to the membrane can activate several pathways that will cause cell death	[7, 38, 64]
		*Escherichia coli*		
		*Agrobacterium tumefaciens*		
		*Pseudomonas cichorii*		
		*P. plantarii*		
		*P. glumae*		
		Fungi	Inhibit spore germination and hyphal growth, interaction with fungal membrane lipids resulting in the formation of membrane pores, and leakage of cytoplasmic materials	
		*Botrytis cinerea*		
		*Fusarium solani*		
		*Magnaporthe grisea*		
		*Alternaria alternata*		

LTP protein (lipid transfer proteins)	Wheat	Fungi	Fungal membranes form a pore resulting in an efflux of intracellular ions culminating in cell death	[7]
		*Rhizoctonia solani*		
		*Curvularia lunata*		
		*Alternaria* sp.		
		*Bipolaris oryzae*		
		*Cylindrocladium*		
		*Scoparium*		
		*Botrytis cinerea*		
		*Sarocladium oryzae*		

Kalata B (cyclotide)	*Oldenlandia affinis*	Bacteria	Induces leakage of contents from phospholipid vesicles and forms large pores in lipid bilayers, has lytic ability causing membrane leakage of helminth, and inhibits the development of nematode larvae and motility of adult worms	[7, 65]
		*Staphylococcus aureus*		
		*E. coli*		
		Nematode		
		*Haemonchus contortus*		
		*Trichostrongylus colubriformis*		

Shepherins (glycine- and histidine-rich peptides)	*Capsella bursa-pastoris*	Bacteria	Insertion into the membrane, triggering disruption of lipid bilayer physical integrity, membrane thinning/formation of transient pores, and destabilization of internal membranes, leading to disruption of the endosome	[66, 67]
		*Erwinia herbicola, Escherichia coli,* and *Pseudomonas putida*		
		Fungi		
		*S. cerevisiae*		
		*C. albicans*		
		*Cryptococcus neoformans*		

**Table 2:** Microbe-derived bioactive peptides and their potential antimicrobial applications.

Name of the peptide	Microbial source	Target pathogen	Mode of action	References
Pediocins	*Pediococcus* spp.	Bacteria	Disrupt proton motive force, formation of pores in the cytoplasmic membrane, and cell membrane dysfunction	[69]
		*Listeria monocytogenes*		

Nisin	*Lactococcus lactis*	Bacteria	Pore formation and the inhibition of cell wall biosynthesis	[70–72]
		*Listeria monocytogenes*		
		*Streptococcus aureus*		
		Spore-forming *Bacillus Clostridium* species		

Lacticin 3147	*Lactococcus lactis*	Bacteria	Cell wall disruption and pore formation	[70]
		*Staphylococcus aureus*		
		*Enterococcus faecalis*		
		*Pneumococcus*		
		*Propionibacterium acnes*, *Streptococcus mutans,* and *Listeria monocytogenes*		
		*Bacillus cereus*		

Enterocin A	*Enterococcus faecium*	Bacteria	Interacts with cell wall and cell receptor, membrane permeabilization causes the leakage, and interferes in DNA replication and mRNA synthesis and transcription	[72, 73]
		*L. monocytogenes*		
		*Staphylococcus aureus* and *Salmonella enterica*		

Propionicin	*P. thoenii, P. jensenii,* and *P. freudenreichii*	Bacteria	Unknown	[74, 75]
		*Helicobacter pylori*		
		*Listeria monocytogenes*		
		*Corynebacterium* spp.		
		*Vibrio parahaemolyticus*		
		*Yersinia enterocolitica*		
		*Pseudomonas* spp.,		
		*Saccharomyces* spp.		
		*Aspergillus* spp.		
		Fungi		
		*Aspergillus wentii* and *Apiotrichum curvatum*		
		*Fusarium tricinctum* and *Phialophora gregata*		
		*Candida, Saccharomyces,* and *Scopulariopsis genera*		

Lynronne	Rumen microbiome	Bacteria	Membrane permeabilization pore formation and lysis	[76, 77]
		*Staphylococcus aureus*		
		*Acinetobacter baumannii*		
		*P. aeruginosa*		

Gramicidin S	*Aneurinibacillus migulanus*	Bacteria	Binding of peptides to Gram-negative LPS and their ability to disrupt Gram-negative cell membranes, accumulation of bacterial membrane phospholipids, and pore formation in the cell membrane	[78, 79]
		*E. coli*		
		*Klebsiella pneumoniae* and *Pseudomonas aeruginosa*		
		*Acinetobacter baumannii Staphylococcus aureus*		
		*Enterococcus faecium*		

Gramicidin A	*Bacillus brevis*	Bacteria	Membrane permeabilization, interruption of internal molecular function (DNA and protein functions), formation of hydroxyl free radicals, and the imbalance of NADH metabolism	[80, 81]
		*Staphylococcus aureus*		

Gramicidin A (1)	*Bacillus brevis*	Bacteria	Disrupts the transmembrane ion concentration gradient by forming an ion channel in a lipid bilayer	[82]
		*S. pyogenes* and *S. pneumoniae*		
		*Enterococcus faecalis*		
		*Streptococcus agalactiae*		

Endolysins (phage-derived AMP)	Bacteriophages of *A. baumannii*	Bacteria	Cause hydrolysis of bacterial cell wall	[83]
		*Acinetobacter baumannii*		
		Fungi		
		*Aspergillus fumigatus*		
		*Candida albicans*		

Tyrocidines	*Bacillus aneurinolyticus*	Bacteria	Binds to bacterial membranes and disrupts structural integrity, resulting in bacterial cell death, disruption of the asexual cycle of the parasite and inhibit the respiration of parasitized red cells	[84–87]
		*B. Subtilis*		
		*C. albicans*		
		Parasite		
		*Caenorhabditis elegans*		
		*Plamodium gallinaceum*		

Valinomycin	*Streptomyces cavourensis, S. fulvissimus, S. roseochromogenes,* and *S. griseus*	Bacteria	Degradation of glycolytic ATP affects respiration and disrupts the K^+^ ion gradient across the cell membrane, and the unbalanced distribution of ions in the bacterium causes cell death and fungal cell wall and cell membrane permeabilization	[88–90]
		*Streptococcus faecalis* and *Micrococcus lysodeikticus*		
		*Staphylococcus aureus*		
		Fungi		
		*Candida albicans*		
		*Cryptococcus albidus*		

Aureobasidin A 1 (cyclodepsipeptides)	*Aureobasidium pullulans* R106	Fungi	Affect spore germination rate, germination initiation, polarized growth of germ tube, and elongation rate; inhibit inositolphosphoryl-ceramide (IPC) synthase; inhibition of sphingolipid biosynthesis resulting in loss of intracellular structure and vacuolization; and membrane trafficking which disturbed cell proliferation of the parasite	[91]
		*Penicillium digitatum*		
		*P. italicum, P. expansum, Botrytis cinerea,* and *Monilinia fructicola*		
		*C. albicans, S. cerevisiae,* and *C. neoformans*		
		*A. fumigates, Schizosaccharomyces pombe,* and *A. nidulans*		
		*A. Niger* and *A. oryzae*		
		Parasite		
		*Toxoplasma gondii*		
		*Leishmania amazonensis*		

Colistin (polymyxin E)	*Paenibacillus polymyxa*	Bacteria	Increase the permeability of the bacterial membrane, leading to leakage of the cytoplasmic content and causing cell death, and bind to lipid portion A and neutralize and inhibit vital respiratory enzymes	[92]
		*E. coli* and *A. baumannii*		
		*P. aeruginosa* and *Stenotrophomonas maltophilia*		
		*Enterobacter* spp.		
		*Klebsiella* spp.		
		*Citrobacter* spp.		
		*Salmonella* spp., and *Shigella* spp.		

Omphalitis	*Omphalotus olearius*	Nematodes	Unknown	[93]
		*Meloidogyne incognita* and *Caenorhabditis elegans*		

Daptomycin	*Streptomyces roseosporus*	Bacteria *staphylococci* and *enterococci*	Bacterial cell membrane, causing rapid membrane depolarization and a potassium ion efflux, followed by the arrest of DNA, RNA, and protein synthesis, resulting in bacterial cell death	[94, 95]

Pargamicin A	*Amycolatopsis* sp.	Bacteria	Disruption of the membrane potential, leading to loss of the membrane function	[94, 95]
		*S. aureus* and *E. faecalis*		

Nocardithiocin	*Nocardia pseudobrasiliensis*	Bacteria	Cell membrane or cell wall permeability	[94, 96]
		*Mycobacterium*		
		*Gordonia* species		
		*M. tuberculosis*		

Xylapeptide	*Xylaria* sp.	Bacteria	Unknown	[94]
		*Bacillus subtilis*		
		*B. cereus, B. megaterium,* and *Micrococcus luteus*		
		*S. aureus* and *Shigella castellani*		
		Fungus		
		*C. albicans*		

Lugdunin	*Staphylococcus lugdunensis*	Bacteria	Impairment of membrane integrity or ion transport and proton leakage in synthetic, protein-free membrane vesicles	[94, 97, 98]
		*Staphylococcus aureus* and *vancomycin-resistant Enterococcus*		

Venturamide	*Oscillatoria* sp.	Parasite	Unknown	[94]
		*P. falciparum*		

Xenoamicin A	*Xenorhabdus doucetiae* and *Xenorhabdus mauleonii*	Parasite	Interacts with the cytoplasmic membrane	[94, 99]
		*P. falciparum*		
		*T. brucei rhodesiense*		

Szentiamide	*Xenorhabdus szentirmaii*	Parasite	Interacts with the cytoplasmic membrane	[99]
		*Plasmodium falciparum*		
		*Trypanosoma cruzi*		
		Bacteria		
		*M. luteus*		

Apicidin	*Fusarium semitectum*	Parasite	Inhibit histone deacetylase of parasite	[100, 101]
		*Plasmodium falciparum* and *T. gondii*		
		*Plasmodium berghei*		

Thiostrepton	Streptomyces *azureus* and *Streptomyces laurentii*	Parasite	Inhibition of protein synthesis by proteasome *β* subunits and inhibition of mRNA translation	[100]
		*Plasmodium berghei*		

Amphomycin	*Streptomyces canus*	Parasite	Inhibits the biosynthesis of the glycolipid precursor of glycosylphosphatidylinositol (GPI) protein by which the variant surface glycoproteins (VSGs) are anchored in the membrane of the parasites	[100]
		*Trypanosoma brucei*		
		*T. b. gambiense*		
		*T. b. rhodesiense*		

Leucinostatins (A and B) and alamethicin	*Paecilomyces* spp	Parasite	Pore formation in the membranes and interruption of cellular homeostasis, resulting in the death of the parasite	[100]
		*Trypanosoma brucei*		
		*T. b. brucei* and *T. b. rhodesiense*		

Haloduracin (lantibiotic)	*Bacillus halodurans*	Bacteria	Pore formation, cell membrane attack, and the inhibition of cell wall synthesis	[102]
		*B. anthracis*		
		Vancomycin-resistant *Enterococcus faecium, Bacillus cereus,* and methicillin-resistant		
		*Staphylococcus aureus*		

Albicidin	Xanthomonas albilineans	Bacteria	Inhibit DNA replication, transcription, supercoiling, gene regulation, and catalytic DNA cleavage-religation cycle of the GyrA subunit	[103, 104]
		*Enterobacter aerogenes*		
		*Escherichia coli*		
		*Haemophilus influenza*		
		*Klebsiella pneumonia*		
		*Shigella sonnei* and *Staphylococcus aureus*		

Griselimycin	Streptomyces griseus	Bacteria	Inhibit nucleic acid biosynthesis by sliding clamp of DNA polymerase III	[104, 105]
		*Mycobacterium tuberculosis*		

Colicin E	*Escherichia coli*	Bacteria	Inhibit nucleic acid biosynthesis by cleaving the targeted cell's DNA or tRNA and digests the peptidoglycan precursors, leading to cell death pore formation in the inner membrane and degrade the internal molecular components	[71, 104, 106, 107]
		*Shiga-toxin-producing E. coli*		
		*Enteroinvasive*		
		*E. coli* and *Shigella*		
		*Enterobacter*		
		*Klebsiella* and *Morganella*		
		*Salmonella, Shigella*, and *Yersinia*		

Dudawalamides	*Moorea producens*	Parasite	Unknown	[94]
		*P. falciparum, Trypanosoma cruzi,* and *Leishmania donovani*		

Ambobactin	*Streptomyces ambofaciens*	Bacteria	Target cytoplasmic membrane	[94, 108]
		*Bacillus subtilis*		
		*Escherichia coli*		
		*Erwinia carotovora*		
		*Pseudomonas syringae*		
		Fungi		
		*Ralstonia solanacearum* and *Xanthomonas oryzae*		

Teixobactin	*Eleftheria terrae*	Bacteria	Inhibits bacterial cell wall synthesis by binding to the precursor of peptidoglycan and teichoic acid	[94]
		*S. aureus*		
		*Streptococcus pneumonia, M. tuberculosis, Clostridium difficile,* and *Bacillus anthracis*		

Maribasins	*Bacillus marinus*	Fungi	Unknown	[109]
		*Alternaria solani, Fusarium oxysporum, Rhizoctonia solani,* and *Verticillium albo-atrum*		

Clavariopsins	*Clavariopsin aquatica*	Fungi	Inhibit the synthesis of fungal cell walls	[91]
		*C. albicans, Aspergillus fumigatus,* and *A. niger*		

Anidulafungin	*Aspergillus oryzae*	Fungi	Inhibition on *β*-(1,3)-glucan synthase	[109]
		*Candida*		

GE81112	*Streptomyces* sp.	Bacteria	Inhibit bacterial protein synthesis machinery	[110]
		*S. pneumoniae, E. faecalis, E. coli* and *B. subtilis,* and *S. pyogenes*		

Carmaphycin B	Symploca sp.	Parasite	Targets *Plasmodium* proteasome	[96]
		*Plasmodium falciparum*		

Kakadumycin A	*Streptomyces* sp.	Bacteria	Binding to DNA prevents RNA synthesis	[111]
		*Bacillus anthracis*		
		*Enterococcus faecium, Staphylococcus simulans,* and *Staphylococcus aureus*		
		*S. pneumonia*		
		*Listeria monocytogenes*		
		Parasite		
		*Plasmodium falciparum*		

Fengycins	*Bacillus subtilis*	Fungi	Disrupt the mitochondrial membrane potential, production of reactive oxygen species, and chromatin condensation in fungal hyphal cells resulting in hyphal cell death	[112]
		*Magnaporthe grisea*		
		*Aspergillus niger*		
		*Mucor rouxii*		
		*Rhizopus stolonifer*		
		*Gibberella zeae* and *Fusarium graminearum*		
		*Sclerotinia sclerotiorum*		

## Data Availability

No data were used to support the study.

## References

[1] Corrêa J. A. F., de Melo Nazareth T., Rocha G. F. d., Luciano F. B. (2023). Bioactive antimicrobial peptides from food proteins: perspectives and challenges for controlling foodborne pathogens. *Pathogens*.

[2] Aslam M. Z., Firdos S., Li Z. (2022). Detecting the mechanism of action of antimicrobial peptides by using microscopic detection techniques. *Foods*.

[3] Silva A. R., Guimarães M. S., Rabelo J. (2022). Recent advances in the design of antimicrobial peptide conjugates. *Journal of Materials Chemistry B*.

[4] Ahmadi-Vavsari F., Farmani J., Dehestani A. (2019). Recombinant production of a bioactive peptide from spotless smooth-hound (Mustelus griseus) muscle and characterization of its antioxidant activity. *Molecular Biology Reports*.

[5] Tüysüz B., Cakir O., Sahin E. Bioactive peptides: formation and impact mechanisms.

[6] Akbarian M., Khani A., Eghbalpour S., Uversky V. N. (2022). Bioactive peptides: synthesis, sources, applications, and proposed mechanisms of action. *International Journal of Molecular Sciences*.

[7] Nawrot R., Barylski J., Nowicki G., Broniarczyk J., Buchwald W., Goździcka-Józefiak A. (2014). Plant antimicrobial peptides. *Folia Microbiologica*.

[8] Dini I., De Biasi M.-G., Mancusi A. (2022). An overview of the potentialities of antimicrobial peptides derived from natural sources. *Antibiotics*.

[9] Zaky A. A., Simal-Gandara J., Eun J.-B., Shim J.-H., Abd El-Aty A. (2021). Bioactivities, applications, safety, and health benefits of bioactive peptides from food and by-products: a review. *Frontiers in Nutrition*.

[10] Yadavalli S. S., Carey J. N., Leibman R. S. (2016). Antimicrobial peptides trigger a division block in *Escherichia coli* through stimulation of a signalling system. *Nature Communications*.

[11] Shen P., Ding K., Wang L. (2023). In vitro and in vivo antimicrobial activity of antimicrobial peptide Jelleine-I against foodborne pathogen Listeria monocytogenes. *International Journal of Food Microbiology*.

[12] Bártová V., Bárta J., Jarošová M. (2019). Antifungal and antimicrobial proteins and peptides of potato (Solanum tuberosum L.) tubers and their applications. *Applied Microbiology and Biotechnology*.

[13] Stec B. (2006). Plant thionins–the structural perspective. *Cellular and Molecular Life Sciences*.

[14] Wang S., Sun-Waterhouse D., Neil Waterhouse G. I., Zheng L., Su G., Zhao M. (2021). Effects of food-derived bioactive peptides on cognitive deficits and memory decline in neurodegenerative diseases: a review. *Trends in Food Science & Technology*.

[15] Wu S., Bekhit A. E.-D. A., Wu Q. (2021). Bioactive peptides and gut microbiota: candidates for a novel strategy for reduction and control of neurodegenerative diseases. *Trends in Food Science & Technology*.

[16] Souza J. C. d. (2015). *Expressão heteróloga da defensiva CP-TIONINA II em nicotiana tabacum visando à proteção contra pseudomonas syringae*.

[17] Odintsova T., Korostyleva T., Utkina L. (2014). Wheat antimicrobial peptides. *Vavilov Journal of Genetics and Breeding*.

[18] Shenkarev Z. O., Gizatullina A. K., Finkina E. I. (2014). Heterologous expression and solution structure of defensin from lentil Lens culinaris. *Biochemical and Biophysical Research Communications*.

[19] Park Y., Choi B. H., Kwak J.-S. (2005). Kunitz-type serine protease inhibitor from potato (Solanum tuberosum L. cv. Jopung). *Journal of Agricultural and Food Chemistry*.

[20] Moreno M., Segura A., García‐Olmedo F. (1994). Pseudothionin‐St1, a potato peptide active against potato pathogens. *European Journal of Biochemistry*.

[21] Pelegrini P. B., Franco O. L. (2005). Plant *γ*-thionins: novel insights on the mechanism of action of a multi-functional class of defense proteins. *The International Journal of Biochemistry & Cell Biology*.

[22] Hermosa M., Turra D., Fogliano V., Monte E., Lorito M. (2006). Identification and characterization of potato protease inhibitors able to inhibit pathogenicity and growth of Botrytis cinerea. *Physiological and Molecular Plant Pathology*.

[23] Feldman M. L., Andreu A. B., Korgan S. (2014). PLPKI: a novel serine protease inhibitor as a potential biochemical marker involved in horizontal resistance to *<scp>P</scp>hytophthora infestans*. *Plant Breeding*.

[24] Revina T., Gerasimova N., Kladnitskaya G., Chalenko G., Valueva T. (2008). Effect of proteinaceous proteinase inhibitors from potato tubers on the growth and development of phytopathogenic microorganisms. *Applied Biochemistry and Microbiology*.

[25] Berrocal-Lobo M., Segura A., Moreno M., López G., García-Olmedo F., Molina A. (2002). Snakin-2, an antimicrobial peptide from potato whose gene is locally induced by wounding and responds to pathogen infection. *Plant Physiology*.

[26] Terras F. R., Torrekens S., Van Leuven F. (1993). A new family of basic cysteine-rich plant antifungal proteins from Brassicaceae species. *FEBS Letters*.

[27] Lorito M., Broadway R., Hayes C. (1994). Proteinase inhibitors from plants as a novel class of fungicides. *Molecular Plant-Microbe Interactions*.

[28] Balandín M., Royo J., Gómez E., Muniz L. M., Molina A., Hueros G. (2005). A protective role for the embryo surrounding region of the maize endosperm, as evidenced by the characterisation of ZmESR-6, a defensin gene specifically expressed in this region. *Plant Molecular Biology*.

[29] Sathoff A. E., Samac D. A. (2019). Antibacterial activity of plant defensins. *Molecular Plant-Microbe Interactions*.

[30] Kraszewska J., Beckett M. C., James T. C., Bond U. (2016). Comparative analysis of the antimicrobial activities of plant defensin-like and ultrashort peptides against food-spoiling bacteria. *Applied and Environmental Microbiology*.

[31] Zhang Y., Lewis K. (2006). Fabatins: new antimicrobial plant peptides. *FEMS Microbiology Letters*.

[32] Chen G.-H., Hsu M.-P., Tan C.-H. (2005). Cloning and characterization of a plant defensin VaD1 from azuki bean. *Journal of Agricultural and Food Chemistry*.

[33] Altemimi A., Lakhssassi N., Abu-Ghazaleh A., Lightfoot D. A. (2017). Evaluation of the antimicrobial activities of ultrasonicated spinach leaf extracts using rapd markers and electron microscopy. *Archives of Microbiology*.

[34] Islam K. T., Velivelli S. L., Berg R. H., Oakley B., Shah D. M. (2017). A novel bi-domain plant defensin MtDef5 with potent broad-spectrum antifungal activity binds to multiple phospholipids and forms oligomers. *Scientific Reports*.

[35] Tantong S., Pringsulaka O., Weerawanich K. (2016). Two novel antimicrobial defensins from rice identified by gene coexpression network analyses. *Peptides*.

[36] do Nascimento V. V., Mello É. d. O., Carvalho L. P. (2015). Pv D1 defensin, a plant antimicrobial peptide with inhibitory activity against Leishmania amazonensis. *Bioscience Reports*.

[37] Hayes B. M., Bleackley M. R., Wiltshire J. L., Anderson M. A., Traven A., van der Weerden N. L. (2013). Identification and mechanism of action of the plant defensin NaD1 as a new member of the antifungal drug arsenal against Candida albicans. *Antimicrobial Agents and Chemotherapy*.

[38] Barbosa Pelegrini P., Del Sarto R. P., Silva O. N., Franco O. L., Grossi-de-Sa M. F. (2011). Antibacterial peptides from plants: what they are and how they probably work. *Biochemistry research international*.

[39] Wong J. H., Ng T. (2006). Limenin, a defensin‐like peptide with multiple exploitable activities from shelf beans. *Journal of Peptide Science*.

[40] Osborn R. W., De Samblanx G. W., Thevissen K. (1995). Isolation and characterisation of plant defensins from seeds of asteraceae, fabaceae, hippocastanaceae and saxifragaceae. *FEBS Letters*.

[41] Barkhuizen H. (2013). *Mode of Action Studies of Defensin Peptides from Native South African Brassicaceae Species*.

[42] Florack D. (1994). *Application of Hordothionins and Cecropin B for Engineering Bacterial Disease Resistance into Plants*.

[43] Murad A. M., Pelegrini P. B., Neto S. M., Franco O. L. (2007). Novel findings of defensins and their utilization in construction of transgenic plants. *Transgenic Plant Journal*.

[44] Schmidt M., Arendt E. K., Thery T. L. (2019). Isolation and characterisation of the antifungal activity of the cowpea defensin Cp-thionin II. *Food Microbiology*.

[45] Sathoff A. E., Velivelli S., Shah D. M., Samac D. A. (2019). Plant defensin peptides have antifungal and antibacterial activity against human and plant pathogens. *Phytopathology*.

[46] Oita S., Ohnishi-Kameyama M., Nagata T. (2000). Binding of barley and wheat *α*-thionins to polysaccharides. *Bioscience Biotechnology and Biochemistry*.

[47] Neto J. X., Pereira M. L., Oliveira J. T. (2017). A chitin-binding protein purified from Moringa oleifera seeds presents anticandidal activity by increasing cell membrane permeability and reactive oxygen species production. *Frontiers in Microbiology*.

[48] Batista A. B., Oliveira J. T., Gifoni J. M. (2014). New insights into the structure and mode of action of Mo-CBP3, an antifungal chitin-binding protein of Moringa oleifera seeds. *PLoS One*.

[49] Wong J. H., Ng T. B. (2005). Lunatusin, a trypsin-stable antimicrobial peptide from lima beans (Phaseolus lunatus L.). *Peptides*.

[50] Yokoyama S., Iida Y., Kawasaki Y., Minami Y., Watanabe K., Yagi F. (2009). The chitin‐binding capability of Cy‐AMP1 from cycad is essential to antifungal activity. *Journal of Peptide Science*.

[51] Wong J. H., Ng T. B. (2005). Vulgarinin, a broad-spectrum antifungal peptide from haricot beans (Phaseolus vulgaris). *The International Journal of Biochemistry & Cell Biology*.

[52] Sharma S., Verma H. N., Sharma N. K. (2014). Cationic bioactive peptide from the seeds of Benincasa hispida. *International Journal of Peptides*.

[53] Ramos M., Souza D., Gomes M. (2014). A phytopathogenic cysteine peptidase from latex of wild rubber vine *Cryptostegia grandiflora*. *The Protein Journal*.

[54] Souza D. P., Freitas C. D., Pereira D. A. (2011). Laticifer proteins play a defensive role against hemibiotrophic and necrotrophic phytopathogens. *Planta*.

[55] Hintz T., Matthews K. K., Di R. (2015). The use of plant antimicrobial compounds for food preservation. *BioMed Research International*.

[56] Wu W.-H., Di R., Matthews K. R. (2013). Antibacterial mode of action of Ib-AMP1 against *Escherichia colin* O157: H7. *Probiotics and antimicrobial proteins*.

[57] Odintsova T., Shcherbakova L., Slezina M. (2020). Hevein-like antimicrobial peptides WAMPs: structure–function relationship in antifungal activity and sensitization of plant pathogenic fungi to tebuconazole by WAMP-2-derived peptides. *International Journal of Molecular Sciences*.

[58] Slavokhotova A. A., Naumann T. A., Price N. P. (2014). Novel mode of action of plant defense peptides–hevein‐like antimicrobial peptides from wheat inhibit fungal metalloproteases. *FEBS Journal*.

[59] Kragh K. M., Nielsen J. E., Nielsen K. K., Dreboldt S., Mikkelsen J. D. (1995). Characterization and localization of new antifungal cysteine-rich proteins from*Beta vulgaris*. *Molecular Plant-Microbe Interactions*.

[60] Rivillas-Acevedo L. A., Soriano-García M. (2007). Isolation and biochemical characterization of an antifungal peptide from Amaranthus hypochondriacus seeds. *Journal of Agricultural and Food Chemistry*.

[61] Koo J. C., Lee S. Y., Chun H. J. (1998). Two hevein homologs isolated from the seed of Pharbitis nil L. exhibit potent antifungal activity. *Biochimica et Biophysica Acta (BBA)- Protein Structure and Molecular Enzymology*.

[62] Rogozhin E. A., Oshchepkova Y. I., Odintsova T. I. (2011). Novel antifungal defensins from Nigella sativa L. seeds. *Plant Physiology and Biochemistry*.

[63] Regente M., Giudici A., Villalaín J., Canal L. (2005). The cytotoxic properties of a plant lipid transfer protein involve membrane permeabilization of target cells. *Letters in Applied Microbiology*.

[64] Kiba A., Saitoh H., Nishihara M., Omiya K., Yamamura S. (2003). C-terminal domain of a hevein-like protein from Wasabia japonica has potent antimicrobial activity. *Plant and Cell Physiology*.

[65] Craik D. J. (2012). Host-defense activities of cyclotides. *Toxins*.

[66] Park C. J., Park C. B., Hong S.-S., Lee H.-S., Lee S. Y., Kim S. C. (2000). Characterization and cDNA cloning of two glycine-and histidine-rich antimicrobial peptides from the roots of shepherd’s purse, Capsella bursa-pastoris. *Plant Molecular Biology*.

[67] Remuzgo C., Oewel T. S., Daffre S. (2014). Chemical synthesis, structure–activity relationship, and properties of shepherin I: a fungicidal peptide enriched in glycine-glycine-histidine motifs. *Amino Acids*.

[68] Kumar P., Kizhakkedathu J. N., Straus S. K. (2018). Antimicrobial peptides: diversity, mechanism of action and strategies to improve the activity and biocompatibility in vivo. *Biomolecules*.

[69] Khorshidian N., Khanniri E., Mohammadi M., Mortazavian A. M., Yousefi M. (2021). Antibacterial activity of pediocin and pediocin-producing bacteria against Listeria monocytogenes in meat products. *Frontiers in Microbiology*.

[70] Morgan S. M., O’connor P. M., Cotter P. D., Ross R. P., Hill C. (2005). Sequential actions of the two component peptides of the lantibiotic lacticin 3147 explain its antimicrobial activity at nanomolar concentrations. *Antimicrobial Agents and Chemotherapy*.

[71] Radaic A., de Jesus M. B., Kapila Y. L. (2020). Bacterial anti-microbial peptides and nano-sized drug delivery systems: the state of the art toward improved bacteriocins. *Journal of Controlled Release*.

[72] Santos J. C., Sousa R. C., Otoni C. G. (2018). Nisin and other antimicrobial peptides: production, mechanisms of action, and application in active food packaging. *Innovative Food Science & Emerging Technologies*.

[73] Wu Y., Pang X., Wu Y., Liu X., Zhang X. (2022). Enterocins: classification, synthesis, antibacterial mechanisms and food applications. *Molecules*.

[74] Gwiazdowska D., Trojanowska K. (2006). Antimicrobial activity and stability of partially purified bacteriocins produced by*Propionibacterium freudenreichii*ssp.*freudenreichii*and ssp.*shermanii*. *Le Lait*.

[75] Poonam, Pophaly S. D., Tomar S. K., De S., Singh R. (2012). Multifaceted attributes of dairy propionibacteria: a review. *World Journal of Microbiology and Biotechnology*.

[76] Jayawant E. S., Hutchinson J., Gašparíková D. (2021). Molecular basis of selectivity and activity for the antimicrobial peptide lynronne‐1 informs rational design of peptide with improved activity. *ChemBioChem*.

[77] Mulkern A. J., Oyama L. B., Cookson A. R. (2022). Microbiome-derived antimicrobial peptides offer therapeutic solutions for the treatment of *Pseudomonas aeruginosa* infections. *Npj Biofilms and Microbiomes*.

[78] Kondejewski L. H., Farmer S. W., Wishart D. S., Kay C. M., Hancock R. W., Hodges R. S. (1996). Modulation of structure and antibacterial and hemolytic activity by ring size in cyclic gramicidin S analogs. *Journal of Biological Chemistry*.

[79] Swierstra J., Kapoerchan V., Knijnenburg A., van Belkum A., Overhand M. (2016). Structure, toxicity and antibiotic activity of gramicidin S and derivatives. *European Journal of Clinical Microbiology & Infectious Diseases*.

[80] Fang S.-T., Huang S.-H., Yang C.-H., Liou J.-W., Mani H., Chen Y.-C. (2022). Effects of calcium ions on the antimicrobial activity of gramicidin A. *Biomolecules*.

[81] Liou J.-W., Hung Y.-J., Yang C.-H., Chen Y.-C. (2015). The antimicrobial activity of gramicidin A is associated with hydroxyl radical formation. *PLoS One*.

[82] Takada Y., Itoh H., Paudel A. (2020). Discovery of gramicidin A analogues with altered activities by multidimensional screening of a one-bead-one-compound library. *Nature Communications*.

[83] Nandi A., Yadav R., Singh A. (2022). Phage derived lytic peptides, a secret weapon against Acinetobacter baumannii—an in silico approach. *Frontiers of Medicine*.

[84] Ahn J., Kassees K., Lee T., Manandhar B., Yousif A. (2017). Strategy and tactics for designing analogs: biochemical characterization of the large molecules. *Comprehensive Medicinal Chemistry III*.

[85] Taliaferro L. G., Coulston F., Silverman M. (1944). The antimalarial activity of tyrothricin against Plasmodium gallinaceum. *Journal of Infectious Diseases*.

[86] Troskie A. M., Rautenbach M., Delattin N. (2014). Synergistic activity of the tyrocidines, antimicrobial cyclodecapeptides from Bacillus aneurinolyticus, with amphotericin B and caspofungin against Candida albicans biofilms. *Antimicrobial Agents and Chemotherapy*.

[87] Xiao Q., Pei D. (2007). High-throughput synthesis and screening of cyclic peptide antibiotics. *Journal of Medicinal Chemistry*.

[88] Makarasen A., Reukngam N., Khlaychan P., Chuysinuan P., Isobe M., Techasakul S. (2018). Mode of action and synergistic effect of valinomycin and cereulide with amphotericin B against Candida albicans and Cryptococcus albidus. *Journal de Mycologie Médicale*.

[89] Ryabova I., Gorneva G., Ovchinnikov Y. A. (1975). Effect of valinomycin on ion transport in bacterial cells and on bacterial growth. *Biochimica et Biophysica Acta (BBA)- Biomembranes*.

[90] Wibowo J. T., Kellermann M. Y., Köck M. (2021). Anti-infective and antiviral activity of valinomycin and its analogues from a sea cucumber-associated bacterium, Streptomyces sp. SV 21. *Marine Drugs*.

[91] Maharani R., Sleebs B. E., Hughes A. B. (2015). Macrocyclic N-methylated cyclic peptides and depsipeptides. *Studies in Natural Products Chemistry*.

[92] Poirel L., Jayol A., Nordmann P. (2017). Polymyxins: antibacterial activity, susceptibility testing, and resistance mechanisms encoded by plasmids or chromosomes. *Clinical Microbiology Reviews*.

[93] Mayer A., Anke H., Sterner O. (1997). Omphalotin, a new cyclic peptide with potent nematicidal activity from Omphalotus olearius I. Fermentation and biological activity. *Natural Product Letters*.

[94] Bajaj K. (2019). Natural bioactive cyclic peptides and peptidomimetics. *Studies in Natural Products Chemistry*.

[95] Igarashi M., Sawa R., Kinoshita N. (2008). Pargamicin A, a novel cyclic peptide antibiotic from Amycolatopsis sp. *Journal of Antibiotics*.

[96] Sakai K., Komaki H., Gonoi T. (2015). Identification and functional analysis of the nocardithiocin gene cluster in Nocardia pseudobrasiliensis. *PLoS One*.

[97] Nelson R. G., Rosowsky A. (2001). Dicyclic and tricyclic diaminopyrimidine derivatives as potent inhibitors of Cryptosporidium parvum dihydrofolate reductase: structure-activity and structure-selectivity correlations. *Antimicrobial Agents and Chemotherapy*.

[98] Schilling N. A., Berscheid A., Schumacher J. (2019). Synthetic lugdunin analogues reveal essential structural motifs for antimicrobial action and proton translocation capability. *Angewandte Chemie International Edition*.

[99] Dreyer J., Malan A. P., Dicks L. M. (2018). Bacteria of the genus Xenorhabdus, a novel source of bioactive compounds. *Frontiers in Microbiology*.

[100] Rojas-Pirela M., Kemmerling U., Quiñones W., Michels P. A., Rojas V. (2023). Antimicrobial peptides (AMPs): potential therapeutic strategy against trypanosomiases?. *Biomolecules*.

[101] Von Bargen K. W., Niehaus E.-M., Bergander K., Brun R., Tudzynski B., Humpf H.-U. (2013). Structure elucidation and antimalarial activity of apicidin F: an apicidin-like compound produced by Fusarium fujikuroi. *Journal of Natural Products*.

[102] Oman T. J., Van Der Donk W. A. (2009). Insights into the mode of action of the two-peptide lantibiotic haloduracin. *ACS Chemical Biology*.

[103] Cociancich S., Pesic A., Petras D. (2015). The gyrase inhibitor albicidin consists of p-aminobenzoic acids and cyanoalanine. *Nature Chemical Biology*.

[104] Lin S., Chen X., Chen H., Cai X., Chen X., Wang S. (2023). The bioprospecting of microbial-derived antimicrobial peptides for sustainable agriculture. *Engineering*.

[105] Fredersdorf M., Kurz M., Bauer A. (2017). Conformational analysis of an antibacterial cyclodepsipeptide active against *Mycobacterium tuberculosis* by a combined ROE and RDC analysis. *Chemistry--A European Journal*.

[106] Jin X., Kightlinger W., Kwon Y.-C., Hong S. H. (2018). Rapid production and characterization of antimicrobial colicins using Escherichia coli-based cell-free protein synthesis. *Synthetic Biology*.

[107] Marković K. G., Grujović M. Ž., Koraćević M. G. (2022). Colicins and microcins produced by Enterobacteriaceae: characterization, mode of action, and putative applications. *International Journal of Environmental Research and Public Health*.

[108] Wei S., Zhang W., Ji Z. (2015). Structure and antibacterial activity of ambobactin, a new telomycin-like cyclic depsipeptide antibiotic produced by Streptomyces ambofaciens F3. *Molecules*.

[109] Helmy N. M., Parang K. (2023). Cyclic peptides with antifungal properties derived from bacteria, fungi, plants, and synthetic sources. *Pharmaceuticals*.

[110] Brandi L., Lazzarini A., Cavaletti L. (2006). Novel tetrapeptide inhibitors of bacterial protein synthesis produced by a Streptomyces sp. *Biochemistry*.

[111] Castillo U., Harper J. K., Strobel G. A. (2003). Kakadumycins, novel antibiotics from Streptomyces sp. NRRL 30566, an endophyte of Grevillea pteridifolia. *FEMS Microbiology Letters*.

[112] Zhang L., Sun C. (2018). Fengycins, cyclic lipopeptides from marine Bacillus subtilis strains, kill the plant-pathogenic fungus Magnaporthe grisea by inducing reactive oxygen species production and chromatin condensation. *Applied and Environmental Microbiology*.

[113] Perez Espitia P. J., de Fátima Ferreira Soares N., dos Reis Coimbra J. S., de Andrade N. J., Souza Cruz R., Alves Medeiros E. A. (2012). Bioactive peptides: synthesis, properties, and applications in the packaging and preservation of food. *Comprehensive Reviews in Food Science and Food Safety*.

[114] Rao S., Zang X., Yang Z., Gao L., Yin Y., Fang W. (2016). Soluble expression and purification of the recombinant bioactive peptide precursor BPP-1 in *Escherichia coli* using a cELP-SUMO dual fusion system. *Protein Expression and Purification*.

[115] Antosova Z., Mackova M., Kral V., Macek T. (2009). Therapeutic application of peptides and proteins: parenteral forever?. *Trends in Biotechnology*.

[116] Ingham A. B., Moore R. J. (2007). Recombinant production of antimicrobial peptides in heterologous microbial systems. *Biotechnology and Applied Biochemistry*.

[117] Ozawa A., Cai Y., Lindberg I. (2007). Production of bioactive peptides in an in vitro system. *Analytical Biochemistry*.

[118] Akbarian M., Yousefi R., Moosavi-Movahedi A. A., Ahmad A., Uversky V. N. (2019). Modulating insulin fibrillation using engineered B-chains with mutated C-termini. *Biophysical Journal*.

[119] Bougatef A., Nedjar-Arroume N., Manni L. (2010). Purification and identification of novel antioxidant peptides from enzymatic hydrolysates of sardinelle (Sardinella aurita) by-products proteins. *Food Chemistry*.

[120] Schägger H. (2006). Tricine–sds-page. *Nature Protocols*.

[121] Cheng Y., Wei H., Sun R., Tian Z., Zheng X. (2016). Rapid method for protein quantitation by Bradford assay after elimination of the interference of polysorbate 80. *Analytical Biochemistry*.

[122] Leon Madrazo A., Segura Campos M. R. (2020). Review of antimicrobial peptides as promoters of food safety: limitations and possibilities within the food industry. *Journal of Food Safety*.

[123] Corrêa J. A. F., Santos J. V. G. d., Evangelista A. G., Pinto A. C. S. M., Macedo R. E. F. d., Luciano F. B. (2021). Combined application of phenolic acids and essential oil components against Salmonella Enteritidis and Listeria monocytogenes in vitro and in ready-to-eat cooked ham. *Lwt*.

[124] Guzmán-Rodríguez J. J., López-Gómez R., Suárez-Rodríguez L. M. (2013). Antibacterial activity of defensin PaDef from avocado fruit (Persea americana var. drymifolia) expressed in endothelial cells against *Escherichia coli* and *Staphylococcus aureus*. *BioMed Research International*.

[125] Tailor R. H., Acland D. P., Attenborough S. (1997). A novel family of small cysteine-rich antimicrobial peptides from seed of Impatiens balsaminaIs derived from a single precursor protein. *Journal of Biological Chemistry*.

[126] Pelegrini P., Noronha E., Muniz M. (2006). An antifungal peptide from passion fruit (Passiflora edulis) seeds with similarities to 2S albumin proteins. *Biochimica et Biophysica Acta (BBA)- Proteins & Proteomics*.

[127] Ribeiro S. M., Almeida R. G., Pereira C. A. (2011). Identification of a Passiflora alata Curtis dimeric peptide showing identity with 2S albumins. *Peptides*.

[128] Taveira G. B., Mathias L. S., da Motta O. V. (2014). Thionin‐like peptides from Capsicum annuum fruits with high activity against human pathogenic bacteria and yeasts. *Peptide Science*.

[129] Taveira G. B., Mello É. O., Carvalho A. O. (2017). Antimicrobial activity and mechanism of action of a thionin‐like peptide from Capsicum annuum fruits and combinatorial treatment with fluconazole against Fusarium solani. *Biopolymers*.

[130] Udenigwe C. C., Okolie C. L., Qian H., Ohanenye I. C., Agyei D., Aluko R. E. (2017). Ribulose-1, 5-bisphosphate carboxylase as a sustainable and promising plant source of bioactive peptides for food applications. *Trends in Food Science & Technology*.

[131] Kobbi S., Balti R., Bougatef A. (2015). Antibacterial activity of novel peptides isolated from protein hydrolysates of RuBisCO purified from green juice alfalfa. *Journal of Functional Foods*.

[132] García-Díez J., Saraiva C. (2021). Use of starter cultures in foods from animal origin to improve their safety. *International Journal of Environmental Research and Public Health*.

[133] Kadyan S., Rashmi H., Pradhan D., Kumari A., Chaudhari A., Deshwal G. K. (2021). Effect of lactic acid bacteria and yeast fermentation on antimicrobial, antioxidative and metabolomic profile of naturally carbonated probiotic whey drink. *Lwt*.

[134] Martín I., Rodríguez A., Alía A., Martínez-Blanco M., Lozano-Ojalvo D., Córdoba J. J. (2022). Control of Listeria monocytogenes growth and virulence in a traditional soft cheese model system based on lactic acid bacteria and a whey protein hydrolysate with antimicrobial activity. *International Journal of Food Microbiology*.

[135] Pinto A., Barbosa J., Albano H., Isidro J., Teixeira P. (2020). Screening of bacteriocinogenic lactic acid bacteria and their characterization as potential probiotics. *Microorganisms*.

[136] Siroli L., Patrignani F., Serrazanetti D. I. (2015). Lactic acid bacteria and natural antimicrobials to improve the safety and shelf-life of minimally processed sliced apples and lamb’s lettuce. *Food Microbiology*.

[137] Lafta H., Jarallah E. M., Darwash A. (2014). Antibacterial activity of fermented camel milk using two lactic acid bacteria. *Journal of the University of Bombay*.

[138] Muhialdin B. J., Algboory H. L. (2018). Identification of low molecular weight antimicrobial peptides from Iraqi camel milk fermented with Lactobacillus plantarum. *PharmaNutrition*.

[139] Ali Redha A., Valizadenia H., Siddiqui S. A., Maqsood S. (2022). A state-of-art review on camel milk proteins as an emerging source of bioactive peptides with diverse nutraceutical properties. *Food Chemistry*.

[140] Abdel-Hamid M., Romeih E., Saporito P. (2020). Camel milk whey hydrolysate inhibits growth and biofilm formation of *Pseudomonas aeruginosa* PAO1 and methicillin-resistant *Staphylococcus aureus*. *Food Control*.

[141] Benkerroum N., Mekkaoui M., Bennani N., Hidane K. (2004). Antimicrobial activity of camel’s milk against pathogenic strains of *Escherichia coli* and Listeria monocytogenes. *International Journal of Dairy Technology*.

[142] Arulrajah B., Muhialdin B. J., Zarei M., Hasan H., Saari N. (2020). Lacto-fermented Kenaf (Hibiscus cannabinus L.) seed protein as a source of bioactive peptides and their applications as natural preservatives. *Food Control*.

[143] Castillo-Juárez I., Blancas-Luciano B. E., García-Contreras R., Fernández-Presas A. M. (2022). Antimicrobial peptides properties beyond growth inhibition and bacterial killing. *PeerJ*.

[144] Kuang M., Yu H., Qiao S. (2021). A novel nano-antimicrobial polymer engineered with chitosan nanoparticles and bioactive peptides as promising food biopreservative effective against foodborne pathogen *E. coli* O157-caused epithelial barrier dysfunction and inflammatory responses. *International Journal of Molecular Sciences*.

[145] Amiri S., Rezaei Mokarram R., Sowti Khiabani M., Rezazadeh Bari M., Alizadeh Khaledabad M. (2022). Characterization of antimicrobial peptides produced by Lactobacillus acidophilus LA-5 and Bifidobacterium lactis BB-12 and their inhibitory effect against foodborne pathogens. *Lwt*.

[146] Pereira P. R., Freitas C. S., Paschoalin V. M. (2021). *Saccharomyces cerevisiae* biomass as a source of next‐generation food preservatives: evaluating potential proteins as a source of antimicrobial peptides. *Comprehensive Reviews in Food Science and Food Safety*.

[147] Zhang Q.-Y., Yan Z.-B., Meng Y.-M. (2021). Antimicrobial peptides: mechanism of action, activity and clinical potential. *Military Medical Research*.

[148] Al-Rikabi J. M. F., Majeed K. R., Al-Fekaik D. F. (2022). Bioactive peptides with the inhibitory activity that are produced by lactic acid bacteria; their importance and mechanism. *Texas Journal of Agriculture and Biological Sciences*.

[149] Tagg J. R., Dajani A. S., Wannamaker L. W. (1976). Bacteriocins of gram-positive bacteria. *Bacteriological Reviews*.

[150] Mokoena M. P. (2017). Lactic acid bacteria and their bacteriocins: classification, biosynthesis and applications against uropathogens: a mini-review. *Molecules*.

[151] Bechinger B., Gorr S.-U. (2017). Antimicrobial peptides: mechanisms of action and resistance. *Journal of Dental Research*.

[152] Hong J., Lu X., Deng Z., Xiao S., Yuan B., Yang K. (2019). How melittin inserts into cell membrane: conformational changes, inter-peptide cooperation, and disturbance on the membrane. *Molecules*.

[153] Oliva R., Del Vecchio P., Grimaldi A. (2019). Membrane disintegration by the antimicrobial peptide (P) GKY20: lipid segregation and domain formation. *Physical Chemistry Chemical Physics*.

[154] Quemé‐Peña M., Juhász T., Mihály J. (2019). Manipulating active structure and function of cationic antimicrobial peptide CM15 with the polysulfonated drug suramin: a step closer to in vivo complexity. *ChemBioChem*.

[155] Lee T.-H., N Hall K., Aguilar M.-I. (2015). Antimicrobial peptide structure and mechanism of action: a focus on the role of membrane structure. *Current Topics in Medicinal Chemistry*.

[156] Abrunhosa F., Faria S., Gomes P. (2005). Interaction and lipid-induced conformation of two cecropin− melittin hybrid peptides depend on peptide and membrane composition. *The Journal of Physical Chemistry B*.

[157] Strandberg E., Wadhwani P., Tremouilhac P., Dürr U. H., Ulrich A. S. (2006). Solid-state NMR analysis of the PGLa peptide orientation in DMPC bilayers: structural fidelity of 2H-labels versus high sensitivity of 19F-NMR. *Biophysical Journal*.

[158] Hale J. D., Hancock R. E. (2007). Alternative mechanisms of action of cationic antimicrobial peptides on bacteria. *Expert Review of Anti-infective Therapy*.

[159] Hancock R., Patrzykat A. (2002). Clinical development of cationic antimicrobial peptides: from natural to novel antibiotics. *Current Drug Target-Infectious Disorders*.

[160] Dennison S. R., Morton L. H., Harris F., Phoenix D. A. (2016). Low pH enhances the action of maximin H5 against *Staphylococcus aureus* and helps mediate lysylated phosphatidylglycerol-induced resistance. *Biochemistry*.

[161] Mandal S. M., Khan J., Mahata D. (2017). A self-assembled clavanin A-coated amniotic membrane scaffold for the prevention of biofilm formation by ocular surface fungal pathogens. *Biofouling*.

[162] Fiorentino F., Sauer J. B., Qiu X. (2021). Dynamics of an LPS translocon induced by substrate and an antimicrobial peptide. *Nature Chemical Biology*.

[163] Cardoso M. H., Meneguetti B. T., Costa B. O. (2019). Non-lytic antibacterial peptides that translocate through bacterial membranes to act on intracellular targets. *International Journal of Molecular Sciences*.

[164] Boman H. G., Agerberth B., Boman A. (1993). Mechanisms of action on *Escherichia coli* of cecropin P1 and PR-39, two antibacterial peptides from pig intestine. *Infection and Immunity*.

[165] Graf M., Mardirossian M., Nguyen F. (2017). Proline-rich antimicrobial peptides targeting protein synthesis. *Natural Product Reports*.

[166] Otvos L., O I., Rogers M. E. (2000). Interaction between heat shock proteins and antimicrobial peptides. *Biochemistry*.

[167] Kragol G., Lovas S., Varadi G., Condie B. A., Hoffmann R., Otvos L. (2001). The antibacterial peptide pyrrhocoricin inhibits the ATPase actions of DnaK and prevents chaperone-assisted protein folding. *Biochemistry*.

[168] Lehrer R., Barton A., Daher K. A., Harwig S., Ganz T., Selsted M. E. (1989). Interaction of human defensins with *Escherichia coli*. Mechanism of bactericidal activity. *Journal of Clinical Investigation*.

[169] de Leeuw E., Li C., Zeng P. (2010). Functional interaction of human neutrophil peptide-1 with the cell wall precursor lipid II. *FEBS Letters*.

[170] Dennison S. R., Harris F., Mura M., Phoenix D. A. (2018). An atlas of anionic antimicrobial peptides from amphibians. *Current Protein & Peptide Science*.

[171] Li S., Hao L., Bao W. (2016). A novel short anionic antibacterial peptide isolated from the skin of *Xenopus laevis* with broad antibacterial activity and inhibitory activity against breast cancer cell. *Archives of Microbiology*.

[172] Daliri E. B.-M., Oh D. H., Lee B. H. (2017). Bioactive peptides. *Foods*.

[173] Ennaas N., Hammami R., Beaulieu L., Fliss I. (2015). Purification and characterization of four antibacterial peptides from protamex hydrolysate of Atlantic mackerel (*Scomber scombrus*) by-products. *Biochemical and Biophysical Research Communications*.

[174] Guinane C. M., Kent R. M., Norberg S. (2015). Generation of the antimicrobial peptide caseicin A from casein by hydrolysis with thermolysin enzymes. *International Dairy Journal*.

[175] Memarpoor-Yazdi M., Asoodeh A., Chamani J. (2012). A novel antioxidant and antimicrobial peptide from hen egg white lysozyme hydrolysates. *Journal of Functional Foods*.

[176] Mansour S. C., Pena O. M., Hancock R. E. (2014). Host defense peptides: front-line immunomodulators. *Trends in Immunology*.

[177] Adje E. Y., Balti R., Lecouturier D. (2013). Controlled enzymatic hydrolysis: a new strategy for the discovery of antimicrobial peptides. *Probiotics and antimicrobial proteins*.

[178] Malik E., Dennison S. R., Harris F., Phoenix D. A. (2016). pH dependent antimicrobial peptides and proteins, their mechanisms of action and potential as therapeutic agents. *Pharmaceuticals*.

[179] Ashaolu T. J. (2021). Nanoemulsions for health, food, and cosmetics: a review. *Environmental Chemistry Letters*.

[180] Cai G., Moffitt K., Navone L., Zhang Z., Robins K., Speight R. (2022). Valorisation of keratin waste: controlled pretreatment enhances enzymatic production of antioxidant peptides. *Journal of Environmental Management*.

[181] Martín M.-H. J., Ángel M.-M. M., Aarón S.-L. J., Israel B.-G. (2022). Protein hydrolysates as biostimulants of plant growth and development. *Biostimulants: Exploring Sources and Applications*.

[182] Pan S., Agyei D., Jeevanandam J., Danquah M. K. (2019). Bio-active peptides: role in plant growth and defense. *Natural Bio-Active Compounds: Volume 3: Biotechnology, Bioengineering, and Molecular Approaches*.

[183] Zhao X., Zhang X., Liu D. (2021). Collagen peptides and the related synthetic peptides: a review on improving skin health. *Journal of Functional Foods*.

[184] Akter S., Huq M. A. (2020). Biologically rapid synthesis of silver nanoparticles by Sphingobium sp. MAH-11T and their antibacterial activity and mechanisms investigation against drug-resistant pathogenic microbes. *Artificial Cells, Nanomedicine, and Biotechnology*.

[185] Akter S., Lee S.-Y., Siddiqi M. Z. (2020). Ecofriendly synthesis of silver nanoparticles by Terrabacter humi sp. nov. and their antibacterial application against antibiotic-resistant pathogens. *International Journal of Molecular Sciences*.

[186] Huq M. A. (2020). Biogenic silver nanoparticles synthesized by Lysinibacillus xylanilyticus MAHUQ-40 to control antibiotic-resistant human pathogens Vibrio parahaemolyticus and Salmonella Typhimurium. *Frontiers in Bioengineering and Biotechnology*.

[187] Huq M. A., Akter S. (2021). Bacterial mediated rapid and facile synthesis of silver nanoparticles and their antimicrobial efficacy against pathogenic microorganisms. *Materials*.

[188] Huq M. A., Akter S. (2021). Biosynthesis, characterization and antibacterial application of novel silver nanoparticles against drug resistant pathogenic *Klebsiella pneumoniae* and Salmonella enteritidis. *Molecules*.

[189] Mohammadrezaei M., Navidshad B., Gheisari A., Toghyani M. (2021). Cottonseed meal bioactive peptides as an alternative to antibiotic growth promoters in broiler chicks. *International Journal of Peptide Research and Therapeutics*.

[190] Nazeer N., Uribe-Diaz S., Rodriguez-Lecompte J. C., Ahmed M. (2021). Antimicrobial peptides as an alternative to relieve antimicrobial growth promoters in poultry. *British Poultry Science*.

[191] Silveira R. F., Roque-Borda C. A., Vicente E. F. (2021). Antimicrobial peptides as a feed additive alternative to animal production, food safety and public health implications: an overview. *Animal Nutrition*.

[192] Tkaczewska J. (2020). Peptides and protein hydrolysates as food preservatives and bioactive components of edible films and coatings-A review. *Trends in Food Science & Technology*.

[193] Fan H., Liu H., Zhang Y., Zhang S., Liu T., Wang D. (2022). Review on plant-derived bioactive peptides: biological activities, mechanism of action and utilizations in food development. *Journal of Future Foods*.

